# Using low-cost sensors and GPS to assess spatiotemporal variations in personal exposure to PM_2.5_ in the Washington State Twin Registry

**DOI:** 10.1016/j.envres.2025.122941

**Published:** 2025-09-24

**Authors:** Ningrui Liu, Ally Avery, Elena Austin, John S. Meschke, Nicola K. Beck, Graeme Carvlin, Yisi Liu, Anne V. Moudon, Igor Novosselov, Jeffry H. Shirai, Glen E. Duncan, Edmund Seto

**Affiliations:** a Department of Environmental and Occupational Health Sciences, University of Washington, Seattle, WA, USA; b Department of Nutrition and Exercise Physiology, Washington State University Health Sciences Spokane, Spokane, WA, USA; c Department of Epidemiology and Environmental Health, University of Kentucky, Lexington, KY, USA; d Department of Urban Design and Planning, University of Washington, Seattle, WA, USA; e Department of Mechanical Engineering, University of Washington, Seattle, WA, USA

**Keywords:** PM_2.5_, Personal exposure, Low-cost sensor, GPS, Microenvironment, Spatiotemporal pattern

## Abstract

Epidemiological studies typically rely on exposure assessments based on ambient PM_2.5_ concentrations at participants’ home addresses. However, these approaches neglect personal exposures indoors and across different non-residential microenvironments. To address this problem, our study combined low-cost sensors and GPS to conduct two-week personal PM_2.5_ monitoring in 168 adults recruited from the Washington State Twin Registry between 2018 and 2021. PM_2.5_ mass concentration, size-resolved particle number concentration, temperature, humidity, and GPS coordinates were recorded at 1-min intervals, providing 5,161,737 data points. We used GPS coordinates and a processing algorithm for automatic classification of microenvironments, including seven land use types and vehicles, and time spent indoors/outdoors. The low-cost sensors were calibrated in-situ, using regulatory monitoring data within 600 m of participants’ outdoor measurements (R^2^ = 0.93). A linear mixed model was used to estimate the associations of multiple spatiotemporal factors with personal exposure concentrations. The average PM_2.5_ exposure concentration was 8.1 ± 15.8 μg/m^3^ for all participants. Indoor exposure concentration was higher than outdoor exposure level, and indoor exposure dose contributed 77 % to the total exposure. Exposures in residential and industrial land use had a higher concentration than in other areas, and accounted for 69 % of the total exposure dose. Furthermore, personal exposure concentration was the highest during winter and evening hours, possibly due to cooking and heating-related behaviors. This study demonstrates that personal monitoring can capture spatiotemporal variations in PM_2.5_ exposure more accurately than home-based approaches based on ambient air quality, and suggests opportunities for controlling exposures in certain microenvironments.

## 1. Introduction

Fine particulate matter (PM_2.5_) is associated with multiple adverse health outcomes, such as lung cancer and cardiovascular diseases. Despite concerted emission regulation and public health efforts, air pollution and especially PM_2.5_ levels remain a challenging problem in many countries. The Global Burden of Diseases, Injuries, and Risk Factors Study (GBD) has documented that PM_2.5_ was one of the top risk factors globally in 2021, contributing 8.0 % of the total disability-adjusted life years (DALYs) (GBD, 2021 Risk Factors Collaborators, 2024). It is therefore essential to accurately assess the individual- and population-level exposure to PM_2.5_ to quantify health impacts.

Large epidemiological studies typically rely on geospatial models and data from ambient air quality monitoring stations or remote sensing to obtain the ambient PM_2.5_ concentrations at/near participants’ home addresses, which are then linked to health outcomes (Hvidtfeldt et al., 2019; Ma et al., 2023; Shi et al., 2020, 2021; Thurston et al., 2016; Wyatt et al., 2022; Zhang et al., 2023). The possible reasons for this reliance include the fact that ambient air pollution is commonly regulated in many countries, and such studies can inform policy development. It is also relatively difficult to measure personal exposure. However, these approaches include two assumptions. The first assumption is that outdoor exposure is an unbiased representation of their total exposure which includes both indoor and outdoor exposure. As people spend over 80 % of their lifetime in indoor environments (Duan, 2013; Klepeis et al., 2001), there is important bias introduced by this assumption because of variability in infiltration rates and generation of PM_2.5_ indoors from sources such as cooking, heating, smoking, and cleaning (Hu et al., 2020; Hu and Zhao, 2022; Liu et al., 2023; Lunderberg et al., 2023). The large variation in indoor PM_2.5_ levels can be related to household characteristics, residents’ behavioral characteristics, socio-economic status, and local climate (Ferguson et al., 2020; Hadeed et al., 2021; Liang et al., 2021a; Long et al., 2001). The second assumption is that people spend most of their time at home, so that estimating exposure at the home address results in the best and least biased estimate of total exposure. This assumption fails to account for important time-activity patterns where people usually move and stay in multiple non-residential microenvironments in a day, such as working in an office, having meals in restaurants, and commuting in vehicles. It also fails to account for the important relationship between time-activity patterns and demographic characteristics, including age, gender, employment status, and housing type (Vosoughkhosravi and Jafari, 2024). The indoor PM_2.5_ concentrations in different microenvironments can also vary substantially and be quite different from concentrations in homes (Koehler et al., 2019; Steinle et al., 2015; Buonanno et al., 2014; Zamora et al., 2018). The above evidence suggests that home-based modeling methods may not accurately represent the personal exposures to PM_2.5_ people experience on a daily basis under “real-world” conditions. Some previous studies also found very weak correlations between personal exposure and outdoor PM_2.5_ levels measured at fixed sites (Ma et al., 2020; Park and Kwan, 2017; Avery et al., 2010; Mölter et al., 2012; Brown et al., 2008; Wang et al., 2022), further complicating the use of traditional exposure methods in exposure-health studies.

To address the extent of what is noted above and related problems, researchers have started to equip subjects with newer, low-cost portable/wearable sensors or samplers to obtain personal exposure data (Koehler et al., 2019; Steinle et al., 2015; Ma et al., 2020; Sloan et al., 2016; Pradhan et al., 2024; Chan et al., 2023; Lim et al., 2024; Wangchuk et al., 2015; Chen et al., 2019; Pillarisetti et al., 2019; Branis and Kolomazníková, 2010; He et al., 2022; Zhang et al., 2018; Lui et al., 2024; Borgini et al., 2011; Li et al., 2023; Vanker et al., 2023; Lepeule et al., 2023; Lin et al., 2020; Xu et al., 2024; Barkjohn et al., 2020; Milá et al., 2018; Liu et al., 2019). A few of these studies used the active sampler to collect filter samples of PM_2.5_ across one or more weeks and obtain average exposures over the monitoring period, which were further utilized for component analysis and source apportionment (Chen et al., 2019; Vanker et al., 2023; Xu et al., 2024). Most other studies leveraged low-cost sensors to monitor real-time exposures and apportioned the personal exposure of PM_2.5_ into various microenvironments, such as home, workplace, transit, restaurant, and school, to capture the spatiotemporal variability of exposure levels (Koehler et al., 2019; Steinle et al., 2015; Ma et al., 2020; Sloan et al., 2016; Pradhan et al., 2024; Lim et al., 2024; Wangchuk et al., 2015; Branis and Kolomazníková, 2010; He et al., 2022; Zhang et al., 2018; Li et al., 2023; Lin et al., 2020; Mila et al., 2018).

However, these approaches still have limitations. From the perspective of identifying microenvironments, some studies relied on time-activity diaries and questionnaires, which is burdensome for relatively long time periods (Pradhan et al., 2024; Chan et al., 2023; Wangchuk et al., 2015; Chen et al., 2019; Branis and Kolomazníková, 2010; Zhang et al., 2018; Borgini et al., 2011). The subjective nature of these data may also lead to recall bias, which may misclassify some visited microenvironments and may not record the exposure time in different microenvironments accurately (Elgethun et al., 2007; Kelly et al., 2013). Some studies leveraged the Global Positioning System (GPS) receiver to track subject’s movement, but then manually identified the microenvironments on a map according to the GPS coordinates and validated them with time-activity diaries, if available (Koehler et al., 2019; Steinle et al., 2015; He et al., 2022). Microenvironments often included home, workplace, and school, while other microenvironments, such as parks and restaurants, were usually neglected (Sloan et al., 2016; Pradhan et al., 2024; Lim et al., 2024; Wangchuk et al., 2015; He et al., 2022; Zhang et al., 2018; Milá et al., 2018). Further, personal exposure in workplaces was too broad, ignoring that the workplaces can cover a variety of microenvironments, such as offices, factories, and restaurants, where the PM_2.5_ concentrations can vary substantially. From a temporal perspective, the personal exposure monitoring usually lasted for a relatively short period, i.e., 1–2 days (Ma et al., 2020; Sloan et al., 2016; Pradhan et al., 2024; Wangchuk et al., 2015; Pillarisetti et al., 2019; Zhang et al., 2018; Borgini et al., 2011; Vanker et al., 2023; Lin et al., 2020), with very few covering more than two weeks (Chen et al., 2019; Branis and Kolomazníková, 2010; Barkjohn et al., 2020). Using short monitoring periods is likely due to the high cost of equipment, burden on participants, time, and labor. Additionally, the calibration of these low-cost sensors is an important issue. Many studies on personal exposure to PM_2.5_ collocated the low-cost sensors with ambient regulatory monitoring stations or some gold-standard instruments for a long period to obtain the calibration model before the personal monitoring began (Steinle et al., 2015; Ma et al., 2020; Sloan et al., 2016; Pradhan et al., 2024; Chan et al., 2023; Lim et al., 2024; Branis and Kolomazníková, 2010; He et al., 2022; Borgini et al., 2011). This becomes impractical if there is a large number of low-cost sensors, and challenging when these sensors are used in a different environment with different particle composition. Recently, some in-situ calibration approaches have been proposed to address this problem (Xiang et al., 2020; Wallace et al., 2021; Lin et al., 2017; Malings et al., 2020; Maag et al., 2018). Other personal monitoring studies collocated low-cost sensors with gravimetric filter-based sampling on site, and obtained a correction factor by comparing the integrated PM_2.5_ mass concentration from the sensor with that from the filter sample across the entire monitoring period (Koehler et al., 2019; Pillarisetti et al., 2019; Li et al., 2023; Lepeule et al., 2023; Lin et al., 2020; Mila et al., 2018; Liu et al., 2024). Nevertheless, this approach loses high-resolution real-time information with large variation from the sensors, and only uses the long-term average.

To address these challenges, this study employed low-cost sensors and GPS receivers integrated into a single wearable device to conduct a two-week personal monitoring of PM_2.5_ exposure for 168 adults recruited from the Washington State Twin Registry between 2018 and 2021. The goals of this study were to (1) quantify the in-situ calibrated personal exposure of PM_2.5_ of participants, partitioned across microenvironments identified using an automated spatial merging method, and (2) compare and contrast this personal monitoring approach with the more common approach of assessing exposure based on ambient concentration at the residential location.

## 2. Methods

### 2.1. Study design

Participants were monozygotic (MZ) twins living in Washington, USA, who were recruited from the Washington State Twin Registry (Strachan et al., 2013; Duncan et al., 2019) for a study that investigated associations between personal exposure monitoring (including PM_2.5_ and allergens) and health (Duncan et al., 2018, 2021). The present study only focused on the personal exposure monitoring aspects of the parent study. Exclusion criteria included residence outside of Washington state, living with a co-twin, physical limitations that limited mobility, pregnancy, smoking or regular secondhand exposure to tobacco smoke, and regular use of NSAID medications. A total of 168 adult twins were finally recruited between April 2018 and June 2021. Each participant carried the personal monitoring device for two weeks, as well as a stand-alone GPS monitor (details in Section 2.2). The local Institutional Review Board approved this study, and all participants provided informed consent (WSU IRB #18773). Details for study design are available in Supplementary Material (SM) 1 Section S1.

### 2.2. Data collection

We developed a Portable University of Washington Particle (PUWP) monitor to obtain the personal time and location-specific exposure to PM_2.5_. The PUWP utilizes a low-cost optical light-scattering real-time particle sensor (Plantower PMS A003), which provides multiple channels of particle measurement information. Counts of particles for six size bins (i.e., >0.3 μm, >0.5 μm, >1 μm, >2.5 μm, >5 μm, and >10 μm) were logged at 1-min intervals. The number concentrations from this sensor have been validated to have a good linear relationship with the reference instrument, and have been applied to indoor, outdoor, and personal monitoring (He et al., 2022; Huang et al., 2021a; Zamora et al., 2019; Zusman et al., 2020). The Plantower sensor additionally provides the mass concentration of particles under atmospheric environment with three size bins (i.e., <1 μm, <2.5 μm, and <10 μm), based on the particle number concentrations (PNC) and the sensor manufacturer’s proprietary algorithm. The PUWP was also equipped with sensors for temperature and relative humidity (Honeywell HIH6131–021-001) and a GPS receiver (Adafruit 790 Ultimate GPS Module with MTK3339 Chipset) to record the real-time coordinates at the same frequency as PM_2.5_ monitoring. All monitoring data were time-stamped in UTC and stored in a removable microSD memory card. The model of the PUWP is shown in SM1 Fig. S1. Each participant was asked to continuously wear the PUWP monitor for two weeks. Each pair of twins was required to perform the personal PM_2.5_ monitoring over roughly the same time period (e.g., starting within one week of each other).

### 2.3. Data processing

**Fig. 1.**
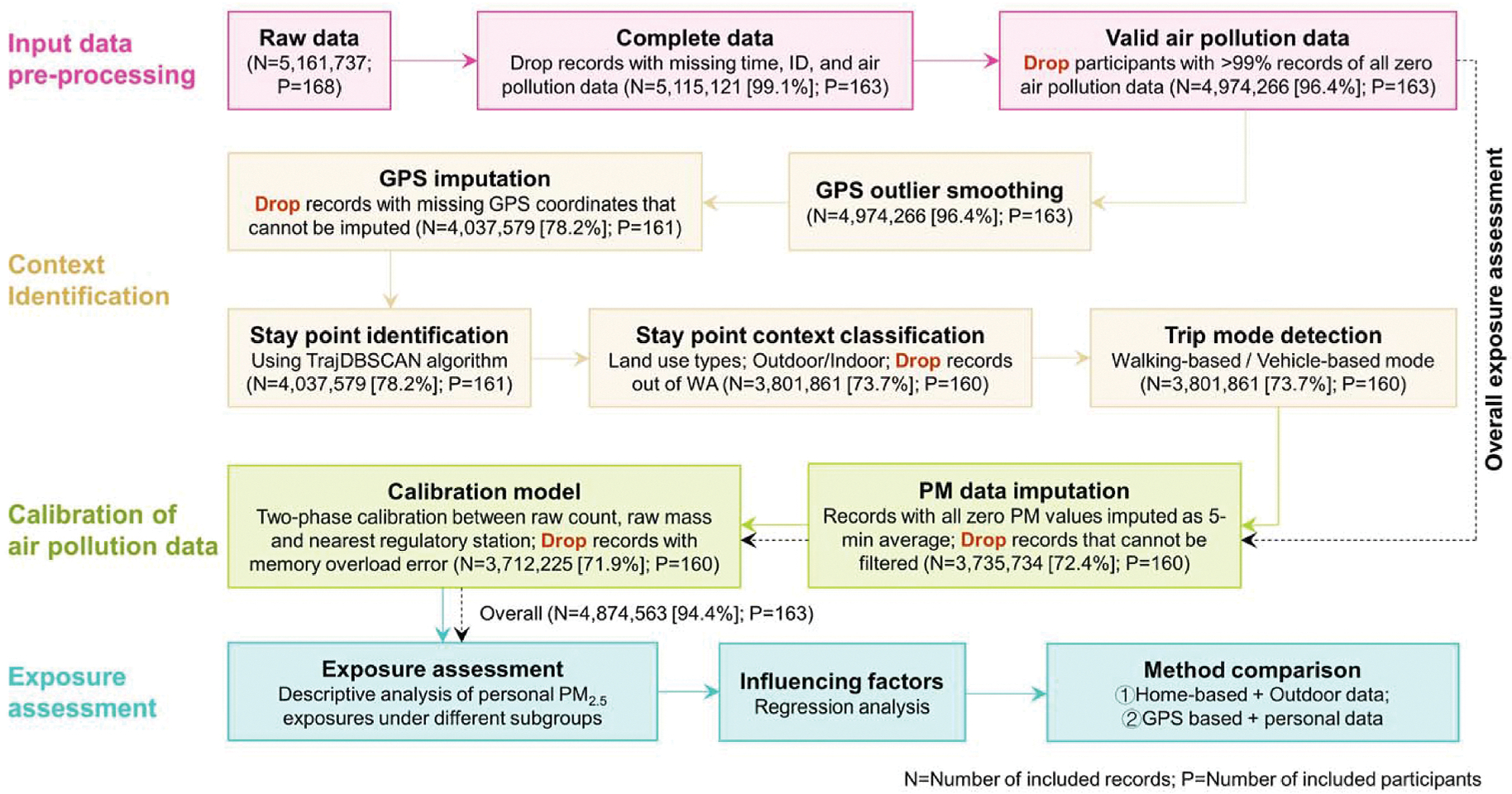
Flowchart of the data processing of personal exposure monitoring.

The data processing procedure is shown in Fig. 1, which was divided into four steps, including data pre-processing, context identification, calibration of air pollution data, and exposure assessment.

#### 2.3.1. Data pre-processing

The raw dataset has 5,161,737 data points in total, collected from 168 participants. We first dropped data points with missing timestamps, ID, or air pollution data (*N* = 46,616, 0.9 %). We then dropped the participants if their PNC and mass concentration were zero values for over 99 % of the monitoring time (*N* = 140,855, 2.7 %). For the overall exposure assessment, the remaining data points (*N* = 4,974,266, 96.4 %) from 163 participants were directly input into the calibration step (Section 2.3.3), rather than through the context identification step (Section 2.3.2).

#### 2.3.2. Context identification

The collected GPS coordinate data were first processed through a moving median filter and missing data imputation (Yi et al., 2022). After excluding GPS data that cannot be imputed, the remaining data points with GPS (*N* = 4,037,579, 78.2 %) were then used to identify the corresponding microenvironments. We first applied the TrajDBSCAN clustering algorithm to classify all data points into different clusters of stay points (i.e., locations where an individual stays for a period of time) and trips (Tran et al., 2010; Zhang and Lin, 2019; Gong et al., 2015; Cheng et al., 2022). Based on land use data from the Washington State Geospatial Portal (Washington State Geospatial Portal Washington, 2010), the land use type of the nearest land use polygon was assigned to each cluster of stay points. We used a 10-m buffer of the building polygons from OpenStreetMap to identify whether the stay points were located in indoor environments. Since study recruitment occurred within Washington state, we excluded those stay points which were out of Washington State (*N* = 235,718, 4.6 %). For data points detected as trips, we followed the method of Yi et al., and used the mean and standard deviation of speed and travel distance for each trip to classify all trips into vehicle-based trips and walking-based trips (Yi et al., 2022). All the vehicle-based trips were assigned as the vehicle microenvironment and considered as indoor activities, while the walking-based trips were assumed to be outdoor and assigned as the land use type of the nearest land use polygon. Details for context identification are available in SM1 Section S2. There was a total of 3,801,861 data points (73.7 %) from 160 participants left after context identification.

#### 2.3.3. Calibration of air pollution data

We used an in-situ calibration approach to compare the hourly average sensor data when the microenvironment context identifies that the sensor is outdoors with the monitoring data from regulatory monitoring stations (Zusman et al., 2020; Stampfer et al., 2020). We assumed that the outdoor PM_2.5_ mass concentration could be estimated using the nearest regulatory monitoring data if the nearest regulatory monitoring station was relatively close to the outdoor sensor location. In this study, we tried different cut-off distances ranging from 0.5 to 5 km, to maximize the goodness-of-fit of the linear mixed model for this calibration. Details for calibration methods are available in SM1 Section S3.

#### 2.3.4. Exposure assessment

According to the parent study, this sub-study focused on personal exposure assessment of PM_2.5_ and did not consider that of size-resolved PNC. Two exposure metrics were used in this section, including exposure concentration and exposure dose. The exposure concentration is the PM_2.5_ mass concentration at a given time (in μg/m^3^), while the exposure dose refers to total mass of PM_2.5_ inhaled during the two-week monitoring period (in μg).

Although data for this study were collected from twin pairs, we assessed individual-level mean hourly PM_2.5_ exposure concentrations during the two-week monitoring period. This part of the analysis did not rely on the microenvironment identification, so we used as many eligible data points as possible (i.e., methods described in Sections 2.3.1 and 2.3.3). A total of 4,874,563 data points (94.4 %) from 163 participants were included. Personal exposure concentrations of PM_2.5_ were compared among different demographic and socioeconomic status (SES) characteristics, including age, sex, race, marital status, highest education level, and annual household income.

Next, we assessed the spatiotemporal patterns of the personal exposures based on microenvironment context identification (methods in Section 2.3.2), with a total of 3,712,225 data points (71.9 %) from 160 participants included. In order to obtain the more accurate time spent in different microenvironments and contribution of each microenvironment to the total exposure, we excluded the invalid participant-days which had less than 6 h for one participant (Yi et al., 2022). From the spatial perspective, personal exposure concentrations of PM_2.5_ in eight microenvironments (including seven land use types and vehicles) and indoor/outdoor environments were summarized across the valid days in the two-week monitoring period and compared with each other. To evaluate the contribution of each microenvironment to the cumulative exposure, the proportion of exposure dose for the *k*th microenvironment was further calculated. From the temporal perspective, we compared the personal PM_2.5_ exposure concentrations in different seasons and hours in a day. Since all two-week personal monitoring were not conducted during the same period in each year, seasonal variation may have a substantial influence on personal PM_2.5_ exposures.

In addition, for comparisons with the personal exposure monitoring data, we assessed the hourly average exposure concentrations at participants’ residential location based only on regulatory monitoring station data, which is an approach used in many epidemiological studies. We applied the inverse distance weighted (IDW) interpolation to obtain the outdoor hourly average PM_2.5_ concentrations at participants’ home addresses from the regulatory monitoring network data (Deng et al., 2015; Pereira et al., 2016; Shukla et al., 2020; Jung et al., 2021; Power et al., 2024). Details for exposure assessment are available in SM1 Section S4.

### 2.4. Statistical analysis

For the descriptive analysis, boxplots and a series of summary statistics, including mean, standard deviation, 2.5th, 25th, 50th, 75th, and 97.5th percentiles, minimum, and maximum, were used to summarize the distribution of personal exposure concentrations overall or in different subgroups across the two-week monitoring period. As the exposure distributions were long-tailed and non-normal, the Wilcoxon test and Kruskal-Wallis test were used to compare the exposure concentrations between two subgroups and across multiple (≥3) subgroups, respectively. If there was a significant difference after the Kruskal-Wallis test, a Bonferroni adjustment was used for the post-hoc pairwise comparison. Furthermore, a linear mixed model was applied to estimate the associations of multiple spatiotemporal factors with personal exposure to PM_2.5_.

(1) $${PM}_{2.5m,\text{calibrated}}\sim \mathrm{Landuse}+IO^{*}{PM}_{2.5m,IDW}+\;\mathrm{Season}\;+\;\mathrm{Hour}+\;\mathrm{Age}\;+\;\mathrm{Sex}\;+\;\mathrm{Marital}\;+\;\mathrm{Race}\;+\;\mathrm{Edu}\;+\;\mathrm{Income}\;+(1|\mathrm{studyid})$$

where Landuse represents seven dummy variables for land use types (reference: other land use); IO is the dummy variable for indoor or outdoor environments (reference: outdoor); ${PM}_{2.5m,IDW}$ is the estimated outdoor PM_2.5_ concentrations at participants’ home addresses through IDW interpolation, which represents the regional background concentration for this participant, μg/m^3^; Season represents three dummy variables for seasons (reference: spring); Hour represents 23 dummy variables for hours in a day (reference: 0 o’clock); Age represents four dummy variables for age groups (reference: 0–29 years old); Sex is the dummy variable for sex (reference: female); Marital is the dummy variable for marital status (reference: unmarried); Race is the dummy variable for race (reference: non-white); Edu represents two dummy variables for highest education level (reference: lower than bachelor (BA)); and Income is the dummy variable for annual household income (reference: low income). To better interpret the possible impacts of all categorical variables in the above model, the estimated marginal means (EMMs) were used for each level of each categorical variable (Searle et al., 1980). The 95 % confidence intervals of coefficients in the linear mixed model were obtained through the profile likelihood approach (Snijders and Bosker, 2011), and *p*-values were adjusted using Benjamini-Hochberg (BH) correction to control the false discovery rate (FDR). To test the robustness of the regression analysis, we conducted two additional sensitivity analyses. In the first approach, several nested models were compared: Model 1 with all demographic covariates excluded (unadjusted model), Model 2 including age and sex (partially adjusted model), and Model 3 including all demographic covariates (fully adjusted model shown in Equation (1)). In the second approach, all participants were divided into ten groups, and a leave-one-group-out strategy was applied by sequentially refitting the regression model on nine groups while leaving one group out. All data analysis in the “Methods” section were performed in R V4.2.2 software using lme4, lmerTest, sf, sp, geosphere, lubridate, reshape2, and emmeans packages (Pebesma, 2018; Pebesma and Bivand, 2023; Bivand et al., 2013; Pebesma and Bivand, 2005; Bates et al., 2015; Kuznetsova et al., 2017; Hijmans; Grolemond and Wickman, 2011; Wickham, 2007; Lenth), as well as QGIS V3.16.7.

## 3. Results

### 3.1. Overall results of personal PM_2.5_ exposure levels

**Fig. 2.**
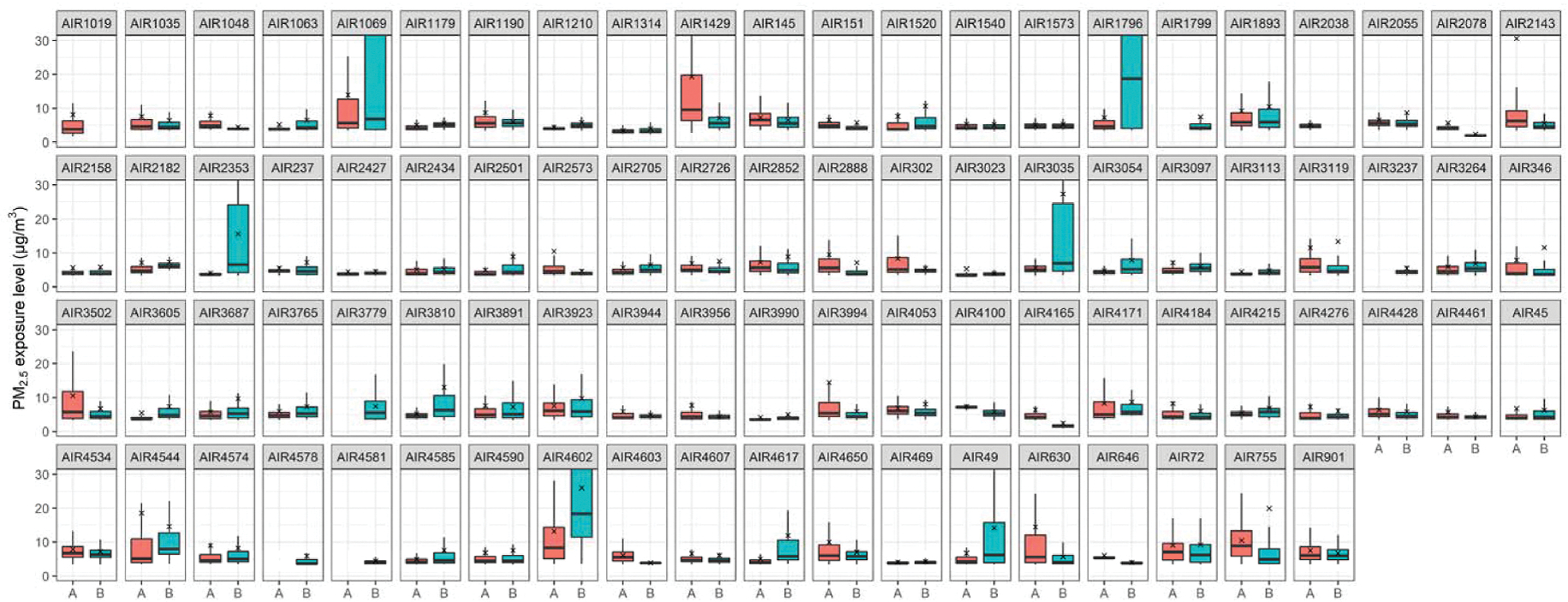
Boxplots of personal PM_2.5_ exposure concentration for all participants grouped by twin pairs.

The calibration results show a high correlation between PM_2.5_ hourly average concentration predictions and those from the nearest regulatory monitoring stations (R^2^ = 0.93, RMSE = 0.1 μg/m^3^), suggesting the high performance of the in-situ calibration in this study (details in SM1 Section S3). After the calibration, the average personal PM_2.5_ exposure concentration of all data points was 8.1 ± 15.8 μg/m^3^, and the median exposure concentration reached 4.6 μg/m^3^. Fig. 2 shows the boxplots of individual-level personal PM_2.5_ exposure concentrations grouped by twin pairs. A total of 163 participants and 78 complete twin pairs were included. Most participants were exposed to PM_2.5_ concentrations lower than 10 μg/m^3^ with relatively small variation, while some participants had a very high PM_2.5_ exposure with substantial variation, such as AIR1069B, AIR1796B, and AIR4602B. Detailed summary statistics of personal exposure concentrations for all participants are provided in Table S7.

The personal PM_2.5_ exposure concentrations were also compared for various demographic and SES characteristics. Figure S8 (a) shows the boxplots of median participant-level exposures. Most medians were lower than 7.5 μg/m^3^. Significant differences were only found between the 50–59 and over 60 years age groups, and between currently married and unmarried participants. The exposure concentrations of participants over 60 years old were lower than the 50–59 age group (difference = 0.9 μg/m^3^). The exposure concentrations of currently married participants were also lower than those of unmarried participants (difference = 0.4 μg/m^3^). Figure S8 (b) provides the boxplots of 97.5th percentiles of participant-level exposures. We found that the 97.5th percentile of exposure concentrations for females were significantly higher than that for males (difference = 5.4 μg/m^3^), while no significant differences were detected for other covariates. Detailed summary statistics can be found in Table S8.

### 3.2. Spatiotemporal patterns of personal PM_2.5_ exposures

In this section, we investigated the possible effect of spatiotemporal factors on personal PM_2.5_ exposure variability. Let’s first consider one participant, AIR4585A, as an example to observe the influence of spatiotemporal factors on PM_2.5_ exposure concentrations. Fig. S9 illustrates the 1-min PM_2.5_ exposure concentration of AIR4585A along with the GPS position for April 23 and April 25 (2018). On April 23, this participant stayed at home in Seattle, Washington, until about 6 a.m. Then, they took a vehicle-based trip to a shopping center in Kirkland, Washington, and finally drove back home around 5:30 p.m. The personal exposure concentrations did not vary a lot during this day and ranged from 3.4 to 7.8 μg/m^3^. The highest exposure concentrations occurred in the shopping center and residence. On April 25, they stayed at home for most of the day, and only drove to a location in Bellevue, Washington, and back home between 4 and 5 p.m. The personal exposure concentration ranged from 3.5 to 26.7 μg/m^3^, and the highest exposure was observed at around 6 a.m. in the residential microenvironment. This example reveals that the large variation in this individual’s personal PM_2.5_ exposures was related to spatiotemporal factors, such as time spent in various microenvironments and time of day.

**Fig. 3.**
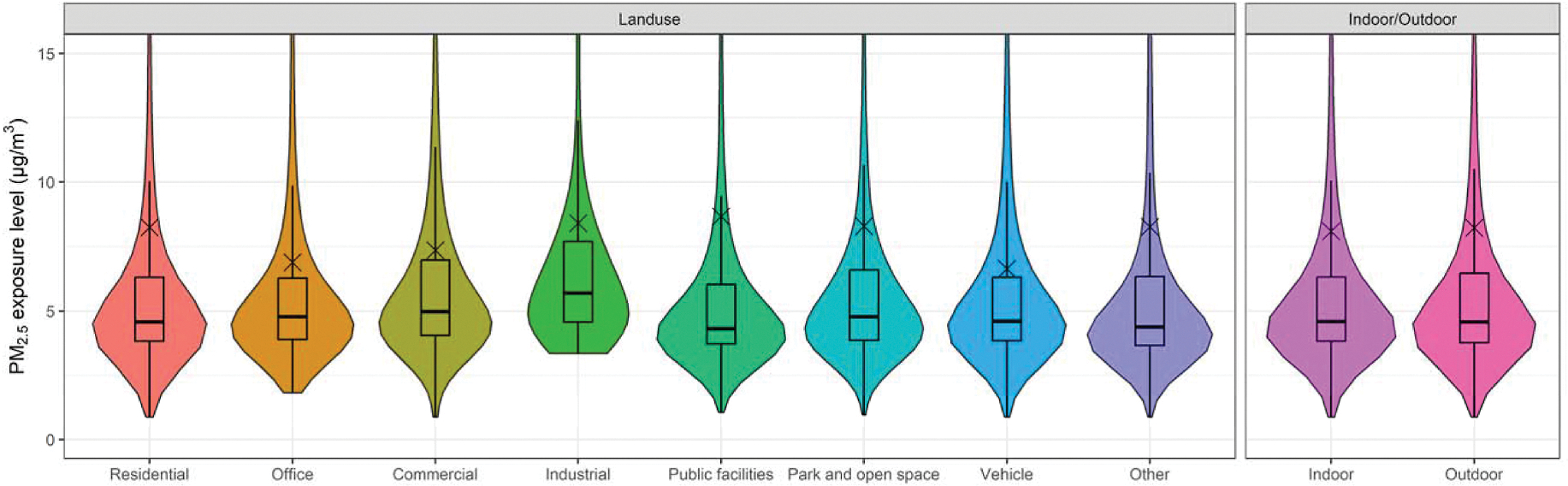
Boxplots of personal PM_2.5_ exposure concentrations in different microenvironments.

**Fig. 4.**
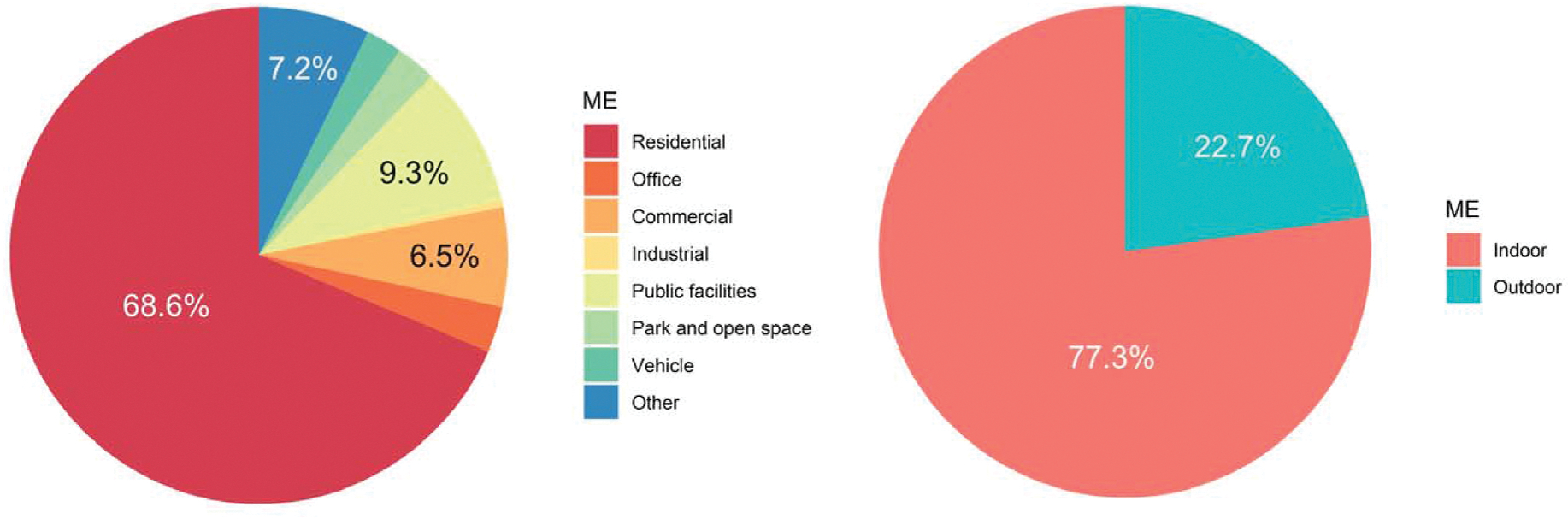
Proportion of total PM_2.5_ exposure dose in different microenvironments.

From the perspective of space, different microenvironments can play a critical role in the spatiotemporal variation in personal PM_2.5_ exposure levels. Fig. S10 shows the time pattern in different microenvironments. Participants spent 78 % of their time in indoor environments, consistent with previous survey results (Duan, 2013; Klepeis et al., 2001). Participants also spent most of their time in residential land use, accounting for 67 % of total time, followed by public facilities (9 %) and commercial land use (7 %). Fig. 3 and Table S9 show the personal exposure concentrations in different microenvironments using the original 3.7 million data points. Differences between all subgroups were found to be significant (*p* < 0.001). The highest median exposure concentration occurred in industrial land use (5.7 μg/m^3^), while the lowest was in public facilities land use (4.3 μg/m^3^). Median indoor exposure concentration was comparable to the median for outdoor exposure (4.6 μg/m^3^). However, all mean values were higher than the 75th percentile, suggesting that these exposure concentrations followed a right-skewed distribution pattern with some extremely high concentrations. For example, although the median exposure concentration in the residential land use was 4.6 μg/m^3^, the mean was 8.3 μg/m^3^ and the proportion of exposure concentrations higher than 20 μg/m^3^ (95th percentile of all data points) reached 5.2 %. Combining the time pattern and exposure concentrations, the contribution of exposure dose in different microenvironments can be further estimated, shown in Fig. 4. Since the exposure concentrations had no substantial difference among various microenvironments, the contribution of exposure dose is nearly proportional to the time spent in each kind of microenvironment. Specifically, indoor exposure dose contributed 77 % to total exposure dose, which was much higher than outdoor exposures. Additionally, the contribution of residential exposure dose ranked first among all land use types and accounted for 69 % of total exposure dose.

**Fig. 5.**
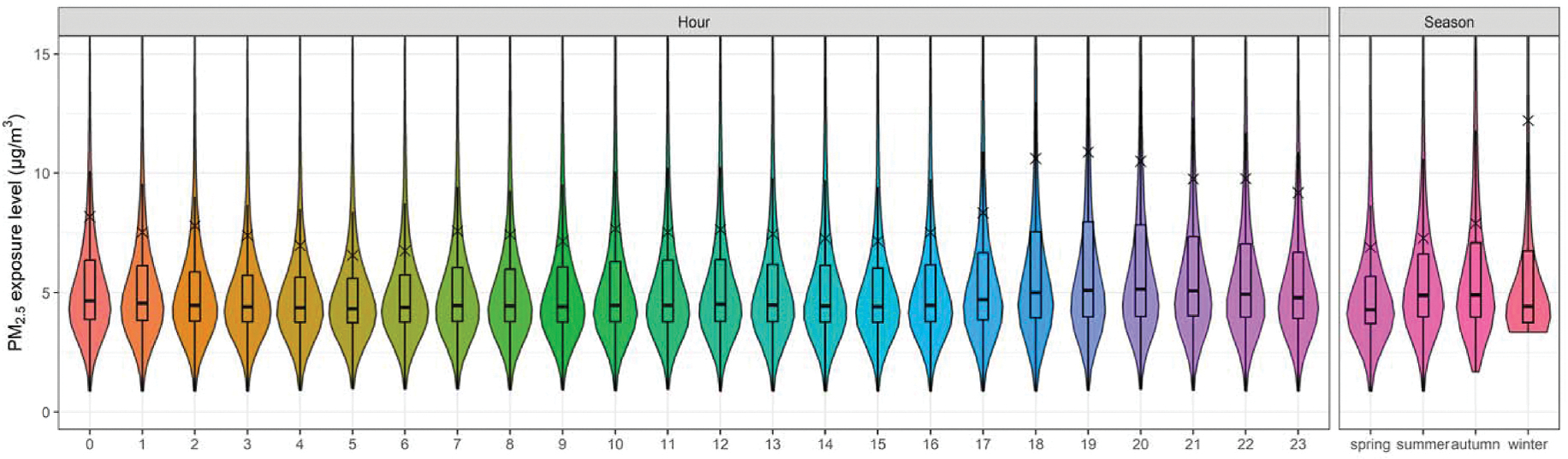
Boxplots of personal PM_2.5_ exposure concentrations in different seasons and hours in a day.

From the perspective of time, personal exposures were compared under two temporal scales, i.e., hourly diurnal patterns and variations across participants monitored in different seasons, as shown in Fig. 5 and Table S10. The personal PM_2.5_ exposure concentration in winter was 12.2 ± 26.3 μg/m^3^, which was higher than other seasons. Diurnal exposure patterns illustrate that for this study, participants’ personal PM_2.5_ exposure tended to rise at 6 p.m. and reach a peak at 7 p.m. (10.9 ± 21.5 μg/m^3^), then gradually decrease until 5 a.m. the next day (6.6 ± 10.8 μg/m^3^, the lowest level).

### 3.3. Comparison with home-based epidemiologic approach

The exposure concentration based on the residential location assessment approach with IDW interpolation of the regulatory monitoring station data was 6.1 ± 7.2 μg/m^3^, which was lower than the GPS-based personal monitoring results (8.1 ± 15.8 μg/m^3^). The variation in exposure concentrations from the residential location approach was also smaller than that in personal exposure concentrations measured in this study. We provide two examples representing two different scenarios to support the above statement. As is shown in Figure S11 (a), the personal exposure concentration had a more dramatic fluctuation in the GPS-based approach, which was influenced by many indoor exposure peaks, than the residential location assessment method. Therefore, the residential location exposure assessment may underestimate the personal exposure method for PM_2.5_. The Pearson correlation between the two results was only 0.28. In contrast, in Figure S11 (b), from August 13 to 15 in 2018, the residential location assessment approach overestimated the personal exposure concentration of the participant. The high outdoor PM_2.5_ concentrations were likely caused by wildfires that occurred during the summer, and reached over 50 μg/m^3^ on some days. However, the personal exposure concentration was lower than 25 μg/m^3^ for most of the time, except for some peaks, leading to a moderate Pearson correlation (r = 0.48). The above evidence demonstrates that high outdoor PM_2.5_ concentrations at home addresses does not necessarily mean high personal exposure concentrations, and vice versa.

### 3.4. Regression analysis of multiple spatiotemporal factors

The subgroup analysis in Section 3.2 and 3.3 considers different influencing factors separately, and does not account for potential confounding. However, spatiotemporal factors along with demographic and SES covariates can affect personal exposure concentrations simultaneously. Table 1 shows the coefficients of the fully adjusted linear mixed model with multiple independent variables. From the perspective of microenvironments, the positive main effect of *IO* suggests that indoor exposure concentration was higher than outdoor exposure by 0.5 μg/m^3^ on average. In addition, the highest PM_2.5_ exposures occurred in the industrial and residential land uses (also illustrated in Fig. S12), higher than the lowest land use (office) by 3.2 and 3.0 μg/m^3^, respectively. The personal exposure in park and open space microenvironments, as well as the vehicle microenvironment, was also relatively low.

From the perspective of time, highest exposure concentration was found in winter, which is consistent with findings in Section 3.2, while the lowest exposure was in summer. The exposures between 7 p.m. and 8 p.m. contributed the highest to total personal PM_2.5_ exposure in a day, while 5 a.m. contributed the lowest. This result agrees well with previous subgroup analysis. Generally, the personal exposure concentration gradually decreased from 7 p.m. to 5 a.m. and from 8 a.m. to 3 p.m.

We included the outdoor concentration at home addresses (PM_2.5m,_ IDW) in this regression model to represent the regional background levels. The positive coefficient of 0.279 suggests that the personal exposure was weakly but positively correlated with the outdoor concentrations. The significant negative interaction between indoor/outdoor environment and regional background PM_2.5_ (−0.168) reveals that the correlation between indoor personal exposure and outdoor concentrations at the home addresses (i.e., 0.279–0.168 = 0.111) was much lower than that between the outdoor personal exposure and outdoor home-based concentrations.

Some demographic and SES covariates were also significant in this regression analysis after adjustment by the above spatiotemporal factors. Participants younger than 30 years old had the highest personal exposure, while those older than 60 years old had the lowest. Exposure concentrations among males were not significantly different from female levels. Unmarried, non-white participants with an education level higher than BA had significantly lower exposure concentrations to PM2.5.

The two sensitivity analyses, including the nested model comparison and leave-one-group-out test, suggest that the coefficients of those spatiotemporal factors are robust enough to support the above findings. Detailed results of the sensitivity analyses can be found in Table S5 and S6.

## 4. Discussion

This study assessed the personal PM_2.5_ exposure levels of 163 participants over two weeks of monitoring, using automatically identified microenvironments and an in-situ calibration approach. The calibration approach showed good performance against the criterion measure (R^2^ = 0.93, RMSE = 0.1 μg/m^3^). Mean (SD) and median (IQR) personal PM_2.5_ exposure concentrations were 8.1 (15.8) μg/m^3^ and 4.6 (2.5) μg/m^3^. Overall, we found that exposure levels varied by certain spatiotemporal characteristics. Notably, based on multivariate regression analysis, indoor exposure concentrations were higher than outdoor exposures, after controlling for other confounding factors. Exposure concentrations that occurred in industrial and residential land uses were higher than in other land use categories. Additionally, personal exposures were the highest during winter and evening hours (around 7 p.m.). Finally, the residential location exposure assessment method based on interpolation of regulatory monitoring station measurements that is used in many epidemiological studies did not capture the spatiotemporal variations in personal PM_2.5_ exposures observed for participants in this study.

Different microenvironments contributed to the large variation in spatiotemporal personal exposure concentration to PM_2.5_. This study found that exposure concentrations occurring in industrial and residential land use were the highest among all microenvironments. A few participants (e.g., AIR1796B, AIR2353B, and AIR3035B) experienced extremely high PM_2.5_ exposure concentrations (>100 μg/m^3^) in their residences during 7 a.m., 10–11 a.m., and 8–10 p.m. (AIR1796B), 5–6 pm (AIR2353B), and 4–6 pm (AIR3035B), respectively. Several previous studies also observed a relatively high exposure concentration in residences compared to other microenvironments (Koehler et al., 2019; Steinle et al., 2015; Wangchuk et al., 2015). Liu et al. demonstrated in their personal monitoring study of pregnant women that there were more peaks and higher peak exposure concentrations in home residential locations (Liu et al., 2024). This is likely due to indoor cooking and heating activities, which is supported by the finding that higher exposures were observed around 7 p.m. (i.e., dinnertime, also found by Koehler et al. (2019)) and winter (i.e., residential heating season). Cooking oil fumes can lead to substantial particle exposures, even with clean fuel (such as electricity), so cooking is an important indoor source of PM_2.5_ which cannot be ignored (Hu and Zhao, 2022; Xiang et al., 2023; Zhai and Albritton, 2020). The EPA Particle TEAM (PTEAM) study measured indoor and outdoor PM_2.5_ concentrations for 178 residents living in Riverside, California in 1990 (Ozkaynak et al., 1996). Combined with questionnaires, they found that cooking contributed 25 % of indoor PM_2.5_ concentrations in 33 homes with cooking activities. Another piece of indirect evidence on the contribution of cooking is that several studies reported relatively high PM_2.5_ exposures in restaurants or eatery microenvironments, which were also likely affected by cooking (Sloan et al., 2016; Li et al., 2023; Lin et al., 2020). For heating, estimates from the 2021 American Community Survey (ACS) show that there are a considerable number of households in Washington State which use wood in their residences (U.S. Census Bureau, 2021). Some field studies have found that solid fuel users are exposed to a significantly higher PM_2.5_ concentration in their residences than clean fuel users (Chan et al., 2023; Lim et al., 2024; Shupler et al., 2020). Together, the above evidence can explain the high exposure concentration in residential land uses, and at 7 p.m. and in winter. We also noted that 70 % of exposure at 7 p.m. with the highest exposure concentration occurred indoors, which further emphasizes the substantial contribution of indoor exposure to the total personal exposure. In addition to cooking and heating, the famous Harvard six-city study and the PTEAM study demonstrated that smoking was an important contributor to indoor and personal PM_2.5_/PM_10_ exposure concentrations (Ozkaynak et al., 1996; Dockery and Spengler, 1981a, 1981b; Sexton et al., 1984; Spengler et al., 1985). However, due to lack of smoking information, the influence of smoking on the variation of PM_2.5_ exposure in this study remains unknown.

In addition to exposure concentration, exposure dose in residential land use is also of great importance. Lin et al. estimated the contribution of microenvironments to the total exposure dose and found that residential exposures accounted for 74.7 % of total exposures across all seasons (Lin et al., 2020), which is very close to the 68.6 % estimate found in this study. Li et al. found that for retired adults in two megacities in China, residential microenvironments accounted for about 85 % of the total PM_2.5_ exposure dose (Li et al., 2023). The slightly higher contribution is likely due to different time-activity patterns between retired people and adults across different age groups in this study. Liu et al. revealed that the peak exposure dose (i.e., area under the curve (AUC) at peaks) in residences was higher than other microenvironments (Liu et al., 2024). Therefore, from the perspective of both exposure level and exposure dose, PM_2.5_ exposure in residential microenvironments should be emphasized, and controlling these exposures should be prioritized in the future.

Ventilation and air purification are commonly used strategies to control residential PM_2.5_ exposure. Previous studies show that using portable air cleaners (PACs) with high-efficiency particulate air (HEPA) filters can substantially reduce the exposure level of PM_2.5_ in households even with uncontrolled ventilation conditions (Huang et al., 2021b; Cooper et al., 2021; Maestas et al., 2019; Barn et al., 2018). Liu et al. focused on different intervention strategies to mitigate cooking-related PM_2.5_ exposure, and found that combining PACs and ventilation was the most effective way to remove cooking-related PM_2.5_ (Liu et al., 2022). They also suggested that adding a stove hood was very helpful in reducing PM_2.5_ concentrations due to cooking (Liu et al., 2022). Some studies further considered both health benefits due to exposure reduction and costs to provide the optimal control approach, such as the best ventilation rate and concentration threshold for PACs (Asikainen et al., 2016; Liu et al., 2021; Yang et al., 2024). Source control is another efficient strategy for reducing residential PM_2.5_ levels, which includes avoiding smoking indoors, reducing solid fuel use for cooking and heating, and replacing old stoves with high-efficiency ones (Li et al., 2017). Some studies pointed to the effect of cooking method and oil types on PM_2.5_ emission rates, where pan-frying and stir-frying emit more particles than deep frying, steaming, and boiling, and olive oil generates more particles than other oil types such as peanut and sunflower oil (Buonanno et al., 2009; Chen et al., 2018; Hu et al., 2012). These findings suggest that change of cooking habits can reduce residential PM_2.5_ exposure concentrations.

This study’s findings related to the importance of PM_2.5_ exposures in industrial land use settings have not been well-documented in previous studies. Although some previous personal monitoring studies have investigated personal exposure concentrations in workplaces generally (Koehler et al., 2019; Steinle et al., 2015; Sloan et al., 2016; Lin et al., 2020), they have not specifically focused on industrial land use. Over 60 % of exposure data in the industrial land use in this study happened outdoors, so the high exposure concentrations in industrial areas may be more related to outdoor PM_2.5_ pollution. The outdoor PM_2.5_ concentrations in industrial areas can come from both industrial emissions and freight transport emissions, such as gasoline/diesel vehicles, trains, and ships, which can be supported by inventories and source apportionment studies. The Washington comprehensive emission inventory in 2020 provided the source contributions to PM_2.5_ in King County, which was the most urbanized area in Washington and where most participants were located in this study (Department of Ecology State of, 2020). Industrial/commercial/institutional fuel use, paved and unpaved road dust, on-road mobile sources, point sources, ships, and railroads contributed 8.3 %, 9.1 %, 4.4 %, 0.8 %, 0.6 %, and 0.2 % of outdoor PM_2.5_, respectively. Additionally, a source apportionment study in Beacon Hill (near the industrial district) in Seattle, identified that gasoline/diesel mobile sources and industry contributed 44 % and 7 % to local PM_2.5_ concentrations (Maykut et al., 2003). Another study reported that freight transport (gasoline, diesel, and fuel oil) and industry (metal processing and cement kiln) accounted for 22 % and 11 % at Duwamish site in the industrial district of Seattle (Kim and Hopke, 2008). Secondary nitrate, probably from vehicle emissions, and secondary sulfate, likely from industrial emissions, contributed 24 % and 20 % of PM_2.5_ mass concentrations, respectively (Kim and Hopke, 2008). All above evidence suggest that freight transport and industrial emission contribute to the higher personal PM_2.5_ exposure concentration in industrial land use in this study.

The spatiotemporal factors likely explain the variation in personal exposure to PM_2.5_ within participant, which was so large that it exceeded the between-participant variation. A variance component analysis was performed using a random intercept model (details in SM1 Section S5) (Koehler et al., 2019). The intraclass correlation coefficient (ICC) was only 0.20, suggesting a relatively small between-participant difference, compared to the within-participant variation. On one hand, although the median difference between each twin pair was only 0.5 (95 % UI: 0.0–5.0) μg/m^3^, health impacts of PM_2.5_ are sensitive to these small changes in exposures at the low exposure range. For instance, according to exposure-response relationships from the GBD Study, if PM_2.5_ concentration increases from 5 to 10 μg/m^3^, the relative risk of ischemic heart disease for people 40–44 years old can increase from 1.20 to 1.36, which means a greater than 13 % higher risk of developing these cardiovascular diseases (GBD, 2019 Risk Factors Collaborators, 2020). Therefore, it is still worthwhile to investigate the impact of this small exposure difference on health outcomes in future epidemiological studies. On the other hand, the large within-participant exposure variation implies that the median or mean exposure concentrations cannot fully represent the whole exposure distribution. Future epidemiological studies can consider other summary statistics, such as 75th and 97.5th percentiles, to depict the peak exposures.

In addition to those spatiotemporal factors, associations between personal PM_2.5_ exposure and demographic covariates were identified in both univariate and multivariate analysis. The univariate analysis helps identify subgroups exposed to higher PM_2.5_ levels. Among different age groups, people over 60 years old had the lowest exposure concentration. This is possibly because older participants spend less time indoors (49 % on average) than younger participants (78 % for those under 30 years old), and indoor concentrations were found significantly higher than outdoor concentrations. Therefore, younger individuals might want to pay more attention to the abovementioned control strategies of indoor PM_2.5_, especially in residences. Unmarried participants had higher exposure concentrations than married participants in this study. Although their time spent indoors was very similar (74 % vs 76 %), unmarried participants were found to have a higher land use diversity (Shannon entropy = 0.88 vs 0.70; detailed formula in SM1 Section S4.1). This suggests that unmarried participants had a higher chance of being exposed to other non-residential land use types with high exposure levels, such as industrial land use, and they can change their route to stay away from possible PM_2.5_ sources or wear N95 masks if necessary. In the multivariate regression analysis, the personal PM_2.5_ exposure was significantly associated with age, marital status, race, and education level. However, since the regression coefficients for these factors were adjusted by spatiotemporal factors, their significant subgroup difference cannot be explained by time patterns. Instead, differences in cooking, smoking, or household cleaning habits among subgroups are likely to contribute to the observed variation in PM_2.5_ exposure. Although we did not collect detailed activity information to validate this hypothesis, it highlights the need for subgroup-specific awareness and strategies to reduce exposure during these indoor source activities. We note that the sample size of older and non-white participants in this study was very limited, so their related findings should be interpreted with caution.

A discrepancy between exposure estimates from the personal monitoring approach and the residential location exposure assessment method based on interpolation of regulatory monitoring station measurements was observed in this study. The residential location exposure assessment approach can sometimes miss some exposure peaks. This may include emissions from some microenvironment-specific sources, such as cooking and smoking, which cannot be reflected by the IDW interpolation of outdoor monitoring data. On the other hand, for outdoor wildfire scenarios, the residential location exposure assessment approach was found to overestimate personal exposures. This can possibly be explained by the participant staying in indoor environments throughout the day, while the outdoor PM_2.5_ had a relatively low infiltration factor indoors, or this participant utilized air cleaners. The isolated peaks seen in the personal monitoring method may result from personal behaviors like window-opening behaviors or going outdoors, or some indoor source emissions. Therefore, the residential location exposure assessment approach used in many epidemiological studies, at least in some cases, may not capture spatiotemporal variations in personal exposure and could underestimate the personal exposure concentration (6.1 vs 8.1 μg/m^3^), which may be the source of bias in air pollution epidemiologic studies. Some previous studies also investigated the comparison between personal exposure and outdoor PM_2.5_ concentration at residential locations (Koehler et al., 2019; Ma et al., 2020; Park and Kwan, 2017; Avery et al., 2010). As observed in our study, they also found that the residential location exposure assessment method does not accurately reflect the true exposure concentration. In this study, the median ratio of personal exposure to outdoor residential location-based exposure (denoted as P/O ratio) was 1.08 (IQR: 0.73–1.61), a bit higher than 0.95 (0.79–1.09) and 0.88 (0.69–1.06), values that were reported in studies from two megacities in China (Li et al., 2023). Since the outdoor PM_2.5_ concentration was much lower in Washington, US than in China, the contribution of indoor source emissions played a more important role in the indoor exposures and corresponding personal exposures, which may lead to a higher P/O ratio in this study. The Pearson correlation between personal and outdoor residential location-based exposure was only 0.10, lower than 0.30 from Koehler et al. in Colorado, USA (Koehler et al., 2019), demonstrating a larger difference between the two exposure assessment results in this study. The possible reason for this lower correlation is that we used minute-level data to estimate the correlation while Koehler et al. used daily average level, which smoothed the concentration peaks and fluctuations during each day (Koehler et al., 2019). Similarly, for PM_10_, previous studies found that the personal-outdoor Pearson correlation was relatively low, such as 0.37 in California in the PTEAM study (Ozkaynak et al., 1996); 0.06 in Vermont (Sexton et al., 1984), and 0.07 in Tennessee (Spengler et al., 1985) from the Harvard six-city study. The above exposure errors between personal and residential location-based exposures, which include both Berkson errors and classical errors, can then lead to bias and variance inflation of health effects obtained in epidemiological studies (Setton et al., 2011; Zeger et al., 2000). Kioumourtzoglou et al. proposed a calibration coefficient for health effect estimates from surrogate exposures (i.e., residential location exposure assessment results) based on paired ambient and personal PM_2.5_ monitoring data in nine cities (Kioumourtzoglou et al., 2014). They found that the calibration coefficient was 0.54 (95 % CI: 0.42–0.65), suggesting an underestimation of health risks using the residential location-based approach (Kioumourtzoglou et al., 2014). Therefore, future studies should apply a personal monitoring approach to obtain unbiased estimates of exposure levels and corresponding health effects.

There are several strengths of this study to highlight: First, this study used a novel PUWP monitor which only costs about USD $300 but combines sensors of particle count, temperature, humidity, GPS, and sound. The Plantower sensors for particles in the PUWP monitors are also used in PurpleAir sensors, which have been widely validated and used in several studies (Lunderberg et al., 2023; Wallace et al., 2021, 2022; Malings et al., 2020; Liang et al., 2021b; Wallace, 2023a, 2023b). However, the PurpleAir sensor, which costs USD $100-$300, does not have a GPS sensor or a battery, so it cannot be used for personal monitoring. By contrast, our PUWP monitor strikes a balance between accuracy and cost-effectiveness, especially in the context of personal monitoring. Second, this study combined GPS data with land use and building data to automatically determine the microenvironments at different times for each participant. This approach can substantially save time and labor compared to time-activity diaries and manually identifying microenvironments. Third, this study used a two-week personal monitoring period, which is longer than previous studies that used one or two days of monitoring, and thus can reflect weekly personal exposures more accurately. Fourth, this study classified microenvironments into seven different land use types and a ‘vehicle’ environment, while most previous studies focused on fewer microenvironments, such as home and school. We also abandoned the usage of “workplace” because it is so general as to include multiple land use types with quite different exposure characteristics.

However, there are some limitations of this study that should be noted. First, the number of participants and the two-week monitoring period were still limited, suggesting that it should be cautious to generalize the findings to larger population. Second, while GPS tracks can tell us about the real-time position and the corresponding microenvironments of the participants, the specific activities they are engaged in are still unknown. Hence, we can only infer the possible activities or sources which led to the high exposures, such as indoor cooking or heating. To avoid burdensome time-activity diaries, a few current studies have used wearable cameras to record people’s real-time activities for aiding the exposure assessment (Milá et al., 2018; Elbassuoni et al., 2023; Li et al., 2022; Salmon et al., 2018). Deep learning algorithms can then be applied on the images to identify possible activities in various microenvironments. Source apportionment could also be considered via chemical composition analysis, size-resolved particle monitoring, or multi-pollutant monitoring, to help identify the specific indoor activities (Xu et al., 2024), or through regression on paired indoor and outdoor low-cost sensor data to quantify the outdoor and indoor source contributions (Lunderberg et al., 2023; Wallace et al., 2022). Third, we used 10-m buffers of buildings to distinguish indoor microenvironments from outdoor microenvironments to minimize the effect of GPS measurement errors. However, it cannot completely rule out the probability of misclassification, especially for indoor cases with poor GPS signals. Future studies can compare and apply GPS receivers with higher accuracy, or combine GPS from smartphones and other devices to double check the GPS coordinates. Fourth, limited data points can be used in the calibration in this study, which only covered the relatively low exposure range and might reduce the generalizability of the calibration equation. Future studies can strengthen this in-situ calibration approach by incorporating collocation data with regulatory monitoring station and other stationary low-cost sensor data such as PurpleAir. Fifth, Plantower sensors do not provide information on their detection limits. Wallace et al. estimated the detection limit of calibrated PM_2.5_ mass concentrations of Plantower PMS5003 based on PNC, which reached 1.2 μg/m^3 53^. It suggests that Plantower sensors cannot accurately measure the very low exposure concentrations even after calibration. However, there were only 0.19 % of calibrated PM_2.5_ mass concentration lower than 1.2 μg/m^3^, which is relatively small and does not substantially bias the exposure assessment.

## 5. Conclusions

This study used low-cost sensors to perform two-week personal PM_2.5_ monitoring for 168 adults from the Washington State Twin Registry between 2018 and 2021. We combined GPS information with land use and building data to automate the classification of microenvironments and obtain the spatiotemporal patterns of personal exposure to PM_2.5_. We also developed an in-situ calibration approach for the low-cost sensors and obtained good performance compared to the gold standard (R^2^ = 0.93). The multivariate regression results suggest that indoor exposure concentrations were slightly higher than outdoor exposures, and PM_2.5_ exposure concentrations in residential and industrial land uses were higher than other microenvironments. These two high-exposure scenarios accounted for 77 % and 69 % of the total exposure dose, respectively, which is consistent with previous studies. In addition, winter and the 6–8 pm time block contributed the most to PM_2.5_ exposure, possibly due to indoor cooking and wood combustion for residential heating. Outdoor wildfire events that occurred during a portion of the monitoring period also caused extremely high personal exposures for some participants. Furthermore, this study demonstrates that the residential location exposure assessment approach used in many epidemiological studies cannot accurately reflect the spatiotemporal patterns of personal exposure concentrations, and will likely lead to bias in estimated exposure concentrations. The findings of this study support the use of low-cost sensors and GPS to improve the precision of personal exposure assessment, and reveal specific microenvironments where PM_2.5_ exposures are high (i.e., residential indoor microenvironments) and can be targeted in future interventions to mitigate the deleterious effects of exposures on health. Future studies can also extend this approach to investigate the spatiotemporal variation of personal exposure to size-resolved PNC.

## Supplementary Material

MMC1

MMC2

Appendix A. Supplementary data

Supplementary data to this article can be found online at https://doi.org/10.1016/j.envres.2025.122941.

## Figures and Tables

**Table 1:** Results of the fully adjusted linear mixed model with multiple spatiotemporal covariates.

Variable	Estimated Marginal Mean (μg/m^3^)	Coefficient	95 % Confidence Interval

(Intercept)	–	8.545	(5.581, 11.510) *
IO			
Outdoor	5.800	Ref	Ref
Indoor	5.297	0.520	(0.464, 0.575) *
PM_2.5m,IDW_	–	0.279	(0.275, 0.284) *
IO×PM_2.5m,IDW_	–	−0.168	(−0.173, −0.163) *
Land use type			
Other land use	6.154	Ref	Ref
Commercial	5.830	−0.324	(−0.418, −0.230) *
Industrial	6.726	0.571	(0.329, 0.814) *
Office	3.511	−2.643	(−2.758, −2.528) *
Park and open space	5.391	−0.763	(−0.882, −0.645) *
Public facilities	5.808	−0.346	(−0.438, −0.254) *
Residential	6.511	0.357	(0.278, 0.435) *
Vehicle	4.461	−1.694	(−1.805, −1.582) *
Season			
Spring	6.740	Ref	Ref
Summer	2.835	−3.905	(−4.004, −3.806) *
Autumn	5.429	−1.312	(−1.513, −1.111) *
Winter	7.191	0.451	(0.308, 0.594) *
Hour in a day			
Hour 0	5.712	Ref	Ref
Hour 1	5.014	−0.698	(−0.802, −0.594) *
Hour 2	5.165	−0.547	(−0.651, −0.443) *
Hour 3	4.677	−1.035	(−1.140, −0.931) *
Hour 4	4.264	−1.448	(−1.552, −1.344) *
Hour 5	3.853	−1.859	(−1.963, −1.755) *
Hour 6	4.042	−1.670	(−1.774, −1.566) *
Hour 7	4.881	−0.831	(−0.935, −0.727) *
Hour 8	4.735	−0.977	(−1.082, −0.873) *
Hour 9	4.485	−1.227	(−1.331, −1.123) *
Hour 10	5.018	−0.694	(−0.798, −0.590) *
Hour 11	4.889	−0.823	(−0.927, −0.719) *
Hour 12	5.100	−0.613	(−0.716, −0.509) *
Hour 13	5.001	−0.711	(−0.815, −0.608) *
Hour 14	4.871	−0.841	(−0.945, −0.738) *
Hour 15	4.855	−0.858	(−0.961, −0.754) *
Hour 16	5.195	−0.517	(−0.620, −0.414) *
Hour 17	5.997	0.285	(0.182, 0.387) *
Hour 18	8.097	2.385	(2.282, 2.487) *
Hour 19	8.327	2.614	(2.512, 2.717) *
Hour 20	7.925	2.212	(2.110, 2.315) *
Hour 21	7.190	1.478	(1.375, 1.581) *
Hour 22	7.190	1.477	(1.374, 1.581) *
Hour 23	6.688	0.975	(0.872, 1.079) *
Age group			
0–29 [20]	9.175	Ref	Ref
30–39 [77]	5.058	−4.116	(−7.481, −0.751) *
40–49 [13]	3.962	−5.213	(−9.750, −0.675) *
50–59 [32]	6.460	−2.714	(−6.457, 1.028)
≥60 [9]	3.089	−6.085	(−11.780, −0.391) *
Sex			
Female [120]	6.491	Ref	Ref
Male [31]	4.607	−1.884	(−4.686, 0.918)
Marital status			
Unmarried [62]	5.503	Ref	Ref
Married [89]	5.594	0.091	(0.026, 0.156) *
Race			
Non-white [9]	4.011	Ref	Ref
White [142]	7.087	3.076	(2.884, 3.268) *
Highest education level			
Lower than BA [31]	5.065	Ref	Ref
BA [66]	6.747	1.682	(1.554, 1.809) *
Higher than BA [54]	4.834	−0.231	(−0.375, −0.088) *
Annual household income			
Low [54]	5.535	Ref	Ref
High [97]	5.563	0.028	(−0.036, 0.092)

Notes:

* refers to *p* < 0.05.

Ref means the reference level for the categorical variable. The number in brackets for age group, sex, marital status, race, highest education level, and annual household income refers to the number of participants included in each category.

## Data Availability

Data will be made available on request.

## References

[R1] Asikainen A, Carrer P, Kephalopoulos S, Fernandes ED, Wargocki P, Hänninen O, 2016. Reducing burden of disease from residential indoor air exposures in Europe (HEALTHVENT project). Environ. Health 15, S35. 10.1186/s12940-016-0101-8 PMC4895703 26961383

[R2] Avery CL, Mills KT, Williams R, McGraw KA, Poole C, Smith RL, Whitsel EA, 2010. Estimating error in using ambient PM2.5 concentrations as proxies for personal exposures: a review. Epidemiology 21 (2), 215–223. 20087191 10.1097/EDE.0b013e3181cb41f7 PMC5370079

[R3] Barkjohn KK, Bergin MH, Norris C, Schauer JJ, Zhang YP, Black MY, Hu M, Zhang JF, 2020. Using low-cost sensors to quantify the effects of air filtration on indoor and personal exposure relevant PM2.5 concentrations in Beijing, China. Aerosol Air Qual. Res. 20 (2), 297–313.

[R4] Barn P, Gombojav E, Ochir C, Laagan B, Beejin B, Naidan G, Boldbaatar B, Galsuren J, Byambaa T, Janes C, Janssen PA, Lanphear BP, Takaro T, Venners SA, Webster GM, Yuchi W, Palmer CD, Parsons PJ, Roh YM, Allen RW, 2018. The effect of portable HEPA filter air cleaners on indoor PM2.5 concentrations and second hand tobacco smoke exposure among pregnant women in Ulaanbaatar, Mongolia: the UGAAR randomized controlled trial. Sci. Total Environ. 615, 1379–1389. 29751442 10.1016/j.scitotenv.2017.09.291

[R5] Bates D, Mächler M, Bolker BM, Walker SC, 2015. Fitting linear mixed-effects models using lme4. J. Stat. Software 67 (1), 1–48.

[R6] Bivand R, Pebesma E, Gomez-Rubio V, 2013. Applied Spatial Data Analysis with R (Second Edition. Springer, New York.

[R7] Borgini A, Tittarelli A, Ricci C, Bertoldi M, De Saeger E, Crosignani P, 2011. Personal exposure to PM2.5 among high-school students in Milan and background measurements: the EuroLifeNet study. Atmos. Environ. 45 (25), 4147–4151.

[R8] Branis M, Kolomazníkova J, 2010. Monitoring of long-term personal exposure to finé particulate matter (PM2.5). Air Qual. Atmos. Health 3 (4), 235–243.

[R9] Brown KW, Sarnat JA, Suh HH, Coull BA, Spengler JD, Koutrakis P, 2008. Ambient site, home outdoor and home indoor particulate concentrations as proxies of personal exposures. J. Environ. Monit. 10 (9), 1041–1051. 18728896 10.1039/b805991h PMC3779934

[R10] Buonanno G, Morawska L, Stabile L, 2009. Particle emission factors during cooking activities. Atmos. Environ. 43 (20), 3235–3242.

[R11] Buonanno G, Stabile L, Morawska L, 2014. Personal exposure to ultrafine particles: the influence of time-activity patterns. Sci. Total Environ. 468, 903–907. 24080417 10.1016/j.scitotenv.2013.09.016

[R12] Chan KH, Xia X, Liu C, Kan H, Doherty A, Yim SHL, Wright N, Kartsonaki C, Yang X, Stevens R, Chang X, Sun D, Yu C, Lv J, Li L, Ho KF, Lam KBH, Chen Z, 2023. China Kadoorie Biobank collaborative, g., characterising personal, household, and community PM(2.5) exposure in one urban and two rural communities in China. Sci. Total Environ. 904, 166647. 37647956 10.1016/j.scitotenv.2023.166647 PMC10804935

[R13] Chen C, Zhao YJ, Zhao B, 2018. Emission rates of multiple air pollutants generated from Chinese residential cooking. Environ. Sci. Technol. 52 (3), 1081–1087. 29302961 10.1021/acs.est.7b05600

[R14] Chen XC, Chow JC, Ward TJ, Cao JJ, Lee SC, Watson JG, Lau NC, Yim SHL, Ho KF, 2019. Estimation of personal exposure to fine particles (PM2.5) of ambient origin for healthy adults in Hong Kong. Sci. Total Environ. 654, 514–524. 30447590 10.1016/j.scitotenv.2018.11.088

[R15] Cheng JY, Zhang XF, Luo P, Huang J, Huang JF, 2022. An unsupervised approach for semantic place annotation of trajectories based on the prior probability. Inf. Sci. 607, 1311–1327.

[R16] Cooper E, Wang Y, Stamp S, Burman E, Mumovic D, 2021. Use of portable air purifiers in homes: operating behaviour, effect on indoor PM2.5 and perceived indoor air quality. Build. Environ. 191, 107621.

[R17] Deng QH, Lu C, Norback D, Bornehag CG, Zhang YP, Liu WW, Yuan H, Sundell J, 2015. Early life exposure to ambient air pollution and childhood asthma in China. Environ. Res. 143, 83–92. 26453943 10.1016/j.envres.2015.09.032

[R18] Department of Ecology State of Washington 2020 Washington Comprehensive Emissions Inventory Technical Support Document, 2020.

[R19] Dockery DW, Spengler JD, 1981a. Personal exposure to respirable particulates and sulfates. Japca J Air Waste Ma 31 (2), 153–159. 10.1080/00022470.1981.10465205 7217532

[R20] Dockery DW, Spengler JD, 1981b. Indoor-outdoor relationships of respirable sulfates and particles. Atmos. Environ. 15 (3), 335–343.

[R21] Duan X, 2013. Exposure Factors Handbook of Chinese Population. China Environment Publishing Group, Beijing, China.

[R22] Duncan GE, Seto E, Avery AR, Oie M, Carvlin G, Austin E, Shirai JH, He J, Ockerman B, Novosselov I, 2018. Usability of a personal air pollution monitor: design-feedback iterative cycle study. JMIR Mhealth Uhealth 6 (12), e12023. 30578204 10.2196/12023 PMC6320397

[R23] Duncan GE, Avery AR, Strachan E, Turkheimer E, Tsang S, 2019. The Washington state twin registry: 2019 update. Twin Res. Hum. Genet. 22 (6), 788–793. 31358074 10.1017/thg.2019.36

[R24] Duncan GE, Avery AR, Tsang S, Williams BD, Seto E, 2021. Changes in physical activity levels and mental health during COVID-19: prospective findings among adult twin pairs. PLoS One 16 (11), e0260218. 34807944 10.1371/journal.pone.0260218 PMC8608318

[R25] Elbassuoni S, Ghattas H, El Ati J, Zoughby Y, Semaan A, Akl C, Trabelsi T, Talhouk R, Ben Gharbia H, Shmayssani Z, Mourad A, with SRG, 2023. Capturing children food exposure using wearable cameras and deep learning. PLOS Digit Health 2 (3), e0000211. 36972212 10.1371/journal.pdig.0000211 PMC10042366

[R26] Elgethun K, Yost MG, Fitzpatrick CT, Nyerges TL, Fenske RA, 2007. Comparison of global positioning system (GPS) tracking and parent-report diaries to characterize children’s time-location patterns. J. Expo. Sci. Environ. Epidemiol. 17 (2), 196–206. 16773123 10.1038/sj.jes.7500496

[R27] Ferguson L, Taylor J, Davies M, Shrubsole C, Symonds P, Dimitroulopoulou S, 2020. Exposure to indoor air pollution across socio-economic groups in high-income countries: a scoping review of the literature and a modelling methodology. Environ. Int. 143, 105748. 32629198 10.1016/j.envint.2020.105748 PMC7903144

[R28] GBD 2019 Risk Factors Collaborators, 2020. Global burden of 87 risk factors in 204 countries and territories, 1990–2019: a systematic analysis for the Global Burden of Disease Study 2019. Lancet 396 (10258), 1223–1249. 33069327 10.1016/S0140-6736(20)30752-2 PMC7566194

[R29] GBD 2021 Risk Factors Collaborators, 2024. Global burden and strength of evidence for 88 risk factors in 204 countries and 811 subnational locations, 1990–2021: a systematic analysis for the Global Burden of Disease Study 2021. Lancet 403 (10440), 2162–2203. 38762324 10.1016/S0140-6736(24)00933-4 PMC11120204

[R30] Gong L, Sato H, Yamamoto T, Miwa T, Morikawa T, 2015. Identification of activity stop locations in GPS trajectories by density-based clustering method combined with support vector machines. J Mod Transp 23, 202–213.

[R31] Grolemond G, Wickman H, 2011. Dates and times made easy with lubridate. J. Stat. Software 40 (3), 1–25.

[R32] Hadeed SJ, O’Rourke MK, Canales RA, Joshweseoma L, Sehongva G, Paukgana M, Gonzalez-Figueroa E, Alshammari M, Burgess JL, Harris RB, 2021. Household and behavioral determinants of indoor PM2.5 in a rural solid fuel burning Native American community. Indoor Air 31 (6), 2008–2019. 34235761 10.1111/ina.12904 PMC8530885

[R33] He JY, Huang CH, Yuan NS, Austin E, Seto E, Novosselov I, 2022. Network of low-cost air quality sensors for monitoring indoor, outdoor, and personal PM2.5 exposure in Seattle during the 2020 wildfire season. Atmos. Environ. 285, 119244.

[R34] Hijmans R. Geosphere: spherical trigonometry (R package version 1.5–19). https://github.com/rspatial/geosphere. (Accessed 12 August 2024).

[R35] Hu Y, Zhao B, 2022. Indoor sources strongly contribute to exposure of Chinese urban residents to PM2.5 and NO2. J. Hazard Mater. 426, 127829. 34863567 10.1016/j.jhazmat.2021.127829

[R36] Hu T, Singer BC, Logue JM, 2012. Compilation of Published PM2.5 Emission Rates for Cooking, Candles and Incense for Use in Modeling of Exposures in Residences. Lawrence Berkeley National Lab.

[R37] Hu Y, Yao MY, Liu YM, Zhao B, 2020. Personal exposure to ambient PM2.5, PM10, O3, NO2, and SO2 for different populations in 31 Chinese provinces. Environ. Int. 144, 106018. 32771828 10.1016/j.envint.2020.106018

[R38] Huang CH, He JY, Austin E, Seto E, Novosselov I, 2021a. Assessing the value of complex refractive index and particle density for calibration of low-cost particle matter sensor for size-resolved particle count and PM2.5 measurements. PLoS One 16 (11), e0259745. 34762676 10.1371/journal.pone.0259745 PMC8584671

[R39] Huang CH, Xiang JB, Austin E, Shirai J, Liu YS, Simpson C, Karr CJ, Fyfe-Johnson AL, Larsen TK, Seto E, 2021b. Impacts of using auto-mode portable air cleaner on indoor PM2.5 levels: an intervention study. Build. Environ. 188, 107444.

[R40] Hvidtfeldt UA, Sorensen M, Geels C, Ketzel M, Khan J, Tjonneland A, Overvad K, Brandt J, Raaschou-Nielsen O, 2019. Long-term residential exposure to PM2.5, PM10, black carbon, NO2, and ozone and mortality in a Danish cohort. Environ. Int. 123, 265–272. 30551059 10.1016/j.envint.2018.12.010

[R41] Jung JY, Park JY, Kim YC, Lee H, Kim E, Kim YS, Lee JP, Kim H, 2021. Effects of air pollution on mortality of patients with chronic kidney disease: a large observational cohort study. Sci. Total Environ. 786, 147471. 33971609 10.1016/j.scitotenv.2021.147471

[R42] Kelly P, Krenn P, Titze S, Stopher P, Foster C, 2013. Quantifying the difference between self-reported and global positioning systems-measured journey durations: a systematic. Transp. Rev. 33 (4), 443–459.

[R43] Kim E, Hopke PK, 2008. Source characterization of ambient fine particles at multiple sites in the Seattle area. Atmos. Environ. 42 (24), 6047–6056.

[R44] Kioumourtzoglou MA, Spiegelman D, Szpiro AA, Sheppard L, Kaufman JD, Yanosky JD, Williams R, Laden F, Hong BL, Suh H, 2014. Exposure measurement error in PM2.5 health effects studies: a pooled analysis of eight personal exposure validation studies. Environ. Health 13, 2. 24410940 10.1186/1476-069X-13-2 PMC3922798

[R45] Klepeis NE, Nelson WC, Ott WR, Robinson JP, Tsang AM, Switzer P, Behar JV, Hern SC, Engelmann WH, 2001. The National Human Activity Pattern Survey (NHAPS): a resource for assessing exposure to environmental pollutants. J. Expo. Anal. Environ. Epidemiol. 11 (3), 231–252. 11477521 10.1038/sj.jea.7500165

[R46] Koehler K, Good N, Wilson A, Mölter A, Moore BF, Carpenter T, Peel JL, Volckens J, 2019. The Fort Collins commuter study: variability in personal exposure to air pollutants by microenvironment. Indoor Air 29 (2), 231–241. 30586194 10.1111/ina.12533 PMC6435329

[R47] Kuznetsova A, Brockhoff PB, Christensen RHB, 2017. lmerTest package: tests in linear mixed effects models. J. Stat. Software 82 (13), 1–26.

[R48] Lenth R Emmeans: estimated marginal means, aka least-squares means. R package version 1.8.2. https://CRAN.R-project.org/package=emmeans.

[R49] Lepeule J, Pin I, Boudier A, Quentin J, Lyon-Caen S, Supernant K, Seyve E, Chartier R, Slama R, Siroux V, group S.s., 2023. Pre-natal exposure to NO(2) and PM(2.5) and newborn lung function: an approach based on repeated personal exposure measurements. Environ. Res. 226, 115656. 36906269 10.1016/j.envres.2023.115656

[R50] Li ZS, Wen QM, Zhang RL, 2017. Sources, health effects and control strategies of indoor fine particulate matter (PM2.5): a review. Sci. Total Environ. 586, 610–622. 28216030 10.1016/j.scitotenv.2017.02.029

[R51] Li WY, Long Y, Kwan MP, Liu NR, Li Y, Zhang YY, 2022. Measuring individuals’ mobility-based exposure to neighborhood physical disorder with wearable cameras. Appl. Geogr. 145, 102728.

[R52] Li N, Xu C, Xu D, Liu Z, Li N, Chartier R, Chang J, Wang Q, Li Y, 2023. Personal exposure to PM(2.5) in different microenvironments and activities for retired adults in two megacities, China. Sci. Total Environ. 865, 161118. 36581280 10.1016/j.scitotenv.2022.161118

[R53] Liang DH, Lee WC, Liao JW, Lawrence J, Wolfson JM, Ebelt ST, Kang CM, Koutrakis P, Sarnat JA, 2021a. Estimating climate change-related impacts on outdoor air pollution infiltration. Environ. Res. 196, 110923. 33705771 10.1016/j.envres.2021.110923 PMC8197171

[R54] Liang YT, Sengupta D, Campmier MJ, Lunderberg DM, Apte JS, Goldstein AH, 2021b. Wildfire smoke impacts on indoor air quality assessed using crowdsourced data in California. Proc. Natl. Acad. Sci. USA 118 (36). 10.1073/pnas.2106478118 PMC8433518 34465624

[R55] Lim S, Said B, Zurba L, Mosler G, Addo-Yobo E, Adeyeye OO, Arhin B, Evangelopoulos D, Fapohunda VT, Fortune F, Griffiths CJ, Hlophe S, Kasekete M, Lowther S, Masekela R, Mkutumula E, Mmbaga BT, Mujuru HA, Nantanda R, Mzati Nkhalamba L, Ngocho JS, Ojo OT, Owusu SK, Shaibu S, Ticklay I, Grigg J, Barratt B, 2024. Characterising sources of PM(2.5) exposure for school children with asthma: a personal exposure study across six cities in Sub-Saharan Africa. Lancet Child Adolesc. Health 8 (1), 17–27. 38000380 10.1016/S2352-4642(23)00261-4 PMC10716619

[R56] Lin C, Masey N, Wu H, Jackson M, Carruthers DJ, Reis S, Doherty RM, Beverland IJ, Heal MR, 2017. Practical field calibration of portable monitors for Mobile measurements of multiple air pollutants. Atmosphere-Basel 8 (12), 231.

[R57] Lin C, Hu DY, Jia X, Chen JH, Deng FR, Guo XB, Heal MR, Cowie H, Wilkinson P, Miller MR, Loh M, 2020. The relationship between personal exposure and ambient PM2.5 and black carbon in Beijing. Sci. Total Environ. 737, 139801. 32783824 10.1016/j.scitotenv.2020.139801

[R58] Liu YS, Lan BW, Shirai J, Austin E, Yang CH, Seto E, 2019. Exposures to air pollution and noise from multi-modal commuting in a Chinese city. Int. J. Environ. Res. Publ. Health 16 (14), 2539. 10.3390/ijerph16142539 PMC6679126 31315275

[R59] Liu YM, Zhou B, Wang JH, Zhao B, 2021. Health benefits and cost of using air purifiers to reduce exposure to ambient fine particulate pollution in China. J. Hazard Mater. 414, 125540. 33684813 10.1016/j.jhazmat.2021.125540

[R60] Liu QY, Son YJ, Li LH, Wood N, Senerat AM, Pantelic J, 2022. Healthy home interventions: distribution of PM2.5 emitted during cooking in residential settings. Build. Environ. 207, 108448.

[R61] Liu NR, Liu W, Deng FR, Liu YM, Gao XH, Fang L, Chen ZR, Tang H, Hong SJ, Pan MY, Liu W, Huo XY, Guo KQ, Ruan FF, Zhang WL, Zhao B, Mo JH, Huang C, Su CX, Sun CJ, Zou ZJ, Li H, Sun YX, Qian H, Zheng XH, Zeng XG, Guo JG, Bu ZM, Mandin C, Haenninen O, Ji JS, Weschler LB, Kan HD, Zhao ZH, Zhang YP, 2023. The burden of disease attributable to indoor air pollutants in China from 2000 to 2017. Lancet Planet. Health 7 (11), E900–E911. 37940210 10.1016/S2542-5196(23)00215-2

[R62] Liu Y, Yi L, Xu Y, Cabison J, Eckel SP, Mason TB, Chu D, Lurvey N, Lerner D, Johnston J, Bastain TM, Farzan SF, Breton CV, Dunton GF, Habre R, 2024. Spatial and temporal determinants of particulate matter peak exposures during pregnancy and early postpartum. Environ. Adv. 17, 100557. 39574825 10.1016/j.envadv.2024.100557 PMC11580741

[R63] Long CM, Suh HH, Catalano PJ, Koutrakis P, 2001. Using time- and size-resolved particulate data to quantify indoor penetration and deposition behavior. Environ. Sci. Technol. 35 (10), 2089–2099. 11393992 10.1021/es001477d

[R64] Lui KH, Zhang T, Man CL, Chan CS, Ho SSH, Qu L, Kwok HHL, Kwok TCY, Ho KF, 2024. Personal exposure monitoring of fine and coarse particulate matter using exposure assessment models for elderly residents in Hong Kong. Chemosphere 357, 141975. 38615960 10.1016/j.chemosphere.2024.141975

[R65] Lunderberg DM, Liang YT, Singer BC, Apte JS, Nazaroff WW, Goldstein AH, 2023. Assessing residential PM2.5 concentrations and infiltration factors with high spatiotemporal resolution using crowdsourced sensors. Proc. Natl. Acad. Sci. 120 (50), e2308832120. 38048461 10.1073/pnas.2308832120 PMC10723120

[R66] Ma J, Tao YH, Kwan MP, Chai YW, 2020. Assessing mobility-based real-time air pollution exposure in space and time using smart sensors and GPS trajectories in Beijing. Ann. Assoc. Am. Geogr. 110 (2), 434–448.

[R67] Ma YDY, Su BB, Li DK, Cui FP, Tang LX, Wang JN, Tian YH, Zheng XY, 2023. Air pollution, genetic susceptibility, and the risk of atrial fibrillation: a large prospective cohort study. Proc. Natl. Acad. Sci. 120 (32), e2302708120. 37523535 10.1073/pnas.2302708120 PMC10410743

[R68] Maag B, Zhou ZM, Thiele L, 2018. A survey on sensor calibration in air pollution monitoring deployments. IEEE Internet Things J. 5 (6), 4857–4870.

[R69] Maestas MM, Brook RD, Ziemba RA, Li FY, Crane RC, Klaver ZM, Bard RL, Spino CA, Adar SD, Morishita M, 2019. Reduction of personal PM2.5 exposure via indoor air filtration systems in Detroit: an intervention study. J. Expo. Sci. Environ. Epidemiol. 29 (4), 484–490. 30420725 10.1038/s41370-018-0085-2 PMC7021209

[R70] Malings C, Tanzer R, Hauryliuk A, Saha PK, Robinson AL, Presto AA, Subramanian R, 2020. Fine particle mass monitoring with low-cost sensors: corrections and long-term performance evaluation. Aerosol Sci. Technol. 54 (2), 160–174.

[R71] Maykut NN, Lewtas J, Kim E, Larson TV, 2003. Source apportionment of PM2.5 at an urban IMPROVE site in Seattle, Washington. Environ. Sci. Technol. 37 (22), 5135–5142. 14655699 10.1021/es030370y

[R72] Milá C, Salmon M, Sanchez M, Ambrós A, Bhogadi S, Sreekanth V, Nieuwenhuijsen M, Kinra S, Marshall JD, Tonne C, 2018. When, where, and what? Characterizing personal PM2.5 exposure in periurban India by integrating GPS, wearable camera, and ambient and personal monitoring data. Environ. Sci. Technol. 52 (22), 13481–13490. 30378432 10.1021/acs.est.8b03075

[R73] Mölter A, Lindley S, de Vocht F, Agius R, Kerry G, Johnson K, Ashmore M, Terry A, Dimitroulopoulou S, Simpson A, 2012. Performance of a microenviromental model for estimating personal NO2 exposure in children. Atmos. Environ. 51, 225–233.

[R74] Ozkaynak H, Xue J, Spengler J, Wallace L, Pellizzari E, Jenkins P, 1996. Personal exposure to airborne particles and metals: results from the particle TEAM study in Riverside, California. J. Expo. Anal. Environ. Epidemiol. 6 (1), 57–78. 8777374

[R75] Park YM, Kwan MP, 2017. Individual exposure estimates may be erroneous when spatiotemporal variability of air pollution and human mobility are ignored. Health Place 43, 85–94. 27914271 10.1016/j.healthplace.2016.10.002

[R76] Pebesma E, 2018. Simple features for R: standardized support for spatial vector data. RMA J. 10 (1), 439–446.

[R77] Pebesma E, Bivand R, 2005. Classes and methods for spatial data in R. R. News 5 (2), 9–13.

[R78] Pebesma E, Bivand R, 2023. Spatial Data Science: with Applications in R. Chapman and Hall/CRC, Boca Raton.

[R79] Pereira G, Bracken MB, Bell ML, 2016. Particulate air pollution, fetal growth and gestational length: the influence of residential mobility in pregnancy. Environ. Res. 147, 269–274. 26918840 10.1016/j.envres.2016.02.001 PMC4821760

[R80] Pillarisetti A, Carter E, Rajkumar S, Young BN, Benka-Coker ML, Peel JL, Johnson M, Clark ML, 2019. Measuring personal exposure to fine particulate matter (PM2.5) among rural Honduran women: a field evaluation of the Ultrasonic Personal Aerosol Sampler (UPAS). Environ. Int. 123, 50–53. 30496981 10.1016/j.envint.2018.11.014 PMC6331229

[R81] Power MC, Bennett EE, Lynch KM, Stewart JD, Xu X, Park ES, Smith RL, Vizuete W, Margolis HG, Casanova R, Wallace R, Sheppard L, Ying Q, Serre ML, Szpiro AA, Chen JC, Liao D, Wellenius GA, van Donkelaar A, Yanosky JD, Whitsel E, 2024. Comparison of PM2.5 air pollution exposures and health effects associations using 11 different modeling approaches in the Women’s Health Initiative Memory Study (WHIMS). Environ. Health Perspect. 132 (1), 17003. 38226465 10.1289/EHP12995 PMC10790222

[R82] Pradhan B, Singh K, Jayaratne R, Thompson H, Jagals P, Gucake J, Hilly JJ, Turagabeci A, Morawska L, 2024. Assessing school children’s personal exposure to PM2.5 in Suva, Fiji. Atmos. Environ. 325, 120448.

[R83] Salmon M, Mila C, Bhogadi S, Addanki S, Madhira P, Muddepaka N, Mora A, Sanchez M, Kinra S, Sreekanth V, Doherty A, Marshall JD, Tonne C, 2018. Wearable camera-derived microenvironments in relation to personal exposure to PM2.5. Environ. Int. 117, 300–307. 29778830 10.1016/j.envint.2018.05.021 PMC6024072

[R84] Searle SR, Speed FM, Milliken GA, 1980. Population marginal means in the linear-model - an alternative to least-squares means. Am. Statistician 34 (4), 216–221.

[R85] Setton E, Marshall JD, Brauer M, Lundquist KR, Hystad P, Keller P, Cloutier-Fisher D, 2011. The impact of daily mobility on exposure to traffic-related air pollution and health effect estimates. J. Expo. Sci. Environ. Epidemiol. 21 (1), 42–48. 20588325 10.1038/jes.2010.14

[R86] Sexton K, Spengler JD, Treitman RD, 1984. Personal exposure to respirable particles - a case-study in Waterbury, Vermont. Atmos. Environ. 18 (7), 1385–1398.

[R87] Shi LH, Wu X, Yazdi MD, Braun D, Abu Awad Y, Wei YG, Liu PF, Di Q, Wang Y, Schwartz J, Dominici F, Kioumourtzoglou MA, Zanobetti A, 2020. Long-term effects of PM2.5 on neurological disorders in the American medicare population: a longitudinal cohort study. Lancet Planet. Health 4 (12), E557–E565. 33091388 10.1016/S2542-5196(20)30227-8 PMC7720425

[R88] Shi LH, Steenland K, Li HM, Liu PF, Zhang YH, Lyles RH, Requia WJ, Ilango SD, Chang HH, Wingo T, Weber RJ, Schwartz J, 2021. A national cohort study (2000–2018) of long-term air pollution exposure and incident dementia in older adults in the United States. Nat. Commun. 12 (1), 6754. 34799599 10.1038/s41467-021-27049-2 PMC8604909

[R89] Shukla K, Kumar P, Mann GS, Khare M, 2020. Mapping spatial distribution of particulate matter using kriging and inverse distance weighting at supersites of megacity Delhi. Sustain. Cities Soc. 54, 101997.

[R90] Shupler M, Hystad P, Birch A, Miller-Lionberg D, Jeronimo M, Arku RE, Chu YL, Mushtaha M, Heenan L, Rangarajan S, Seron P, Lanas F, Cazor F, Lopez-Jaramillo P, Camacho PA, Perez M, Yeates K, West N, Ncube T, Ncube B, Chifamba J, Yusuf R, Khan A, Hu B, Liu XY, Wei L, Tse LA, Mohan D, Kumar P, Gupta R, Mohan I, Jayachitra KG, Mony PK, Rammohan K, Nair S, Lakshmi PVM, Sagar V, Khawaja R, Iqbal R, Kazmi K, Yusuf S, Brauer M, 2020. Household and personal air pollution exposure measurements from 120 communities in eight countries: results from the PURE-AIR study. Lancet Planet. Health 4 (10), E451–E462. 33038319 10.1016/S2542-5196(20)30197-2 PMC7591267

[R91] Sloan CD, Philipp TJ, Bradshaw RK, Chronister S, Barber WB, Johnston JD, 2016. Applications of GPS-tracked personal and fixed-location PM2.5 continuous exposure monitoring. J. Air Waste Manag. Assoc. 66 (1), 53–65. 26512925 10.1080/10962247.2015.1108942

[R92] Snijders TAB, Bosker RJ, 2011. Multilevel Analysis: an Introduction to Basic and Advanced Multilevel Modeling. Sage.

[R93] Spengler JD, Treitman RD, Tosteson TD, Mage DT, Soczek ML, 1985. Personal exposures to respirable particulates and implications for air-pollution epidemiology. Environ. Sci. Technol. 19 (8), 700–707. 22166030 10.1021/es00138a008

[R94] Stampfer O, Austin E, Ganuelas T, Fiander T, Seto E, Karr CJ, 2020. Use of low-cost PM monitors and a multi-wavelength aethalometer to characterize PM2.5 in the Yakama Nation reservation. Atmos. Environ. 224, 117292. 10.1016/j.atmosenv.2020.117292 PMC7566892 33071560

[R95] Steinle S, Reis S, Sabel CE, Semple S, Twigg MM, Braban CF, Leeson SR, Heal MR, Harrison D, Lin C, Wu H, 2015. Personal exposure monitoring of PM2.5 in indoor and outdoor microenvironments. Sci. Total Environ. 508, 383–394. 25497678 10.1016/j.scitotenv.2014.12.003

[R96] Strachan E, Hunt C, Afari N, Duncan G, Noonan C, Schur E, Watson N, Goldberg J, Buchwald D, 2013. University of Washington twin registry: poised for the next generation of twin research. Twin Res. Hum. Genet. 16 (1), 455–462. 10.1017/thg.2012.124 PMC4505360 23218177

[R97] Thurston GD, Ahn J, Cromar KR, Shao YZ, Reynolds HR, Jerrett M, Lim CC, Shanley R, Park Y, Hayes RB, 2016. Ambient particulate matter air pollution exposure and mortality in the NIH-AARP diet and health cohort. Environ. Health Perspect. 124 (4), 484–490. 26370657 10.1289/ehp.1509676 PMC4829984

[R98] Tran LH, Nguyen QVH, Do NH, Yan Z, 2010. Robust and Hierarchical Stop Discovery in Sparse and Diverse Trajectories.

[R99] U.S. Census Bureau, 2021. American Community Survey.

[R100] Vanker A, Barnett W, Chartier R, MacGinty R, Zar HJ, 2023. Personal monitoring of fine particulate matter (PM2.5) exposure in mothers and young children in a South African birth cohort study - a pilot study. Atmos. Environ. 294, 119513.

[R101] Vosoughkhosravi S, Jafari A, 2024. Mapping residential occupancy: understanding sociodemographic influences on occupancy patterns using the American Time Use Survey. J. Comput. Civ. Eng. 38 (6), 04024036.

[R102] Wallace L, 2023a. Cracking the code-matching a proprietary algorithm for a low-cost sensor measuring PM1 and PM2.5. Sci. Total Environ. 893, 164874. 37336395 10.1016/j.scitotenv.2023.164874

[R103] Wallace L, 2023b. Testing a new “decrypted” algorithm for plantower sensors measuring PM2.5: Comparison with an alternative algorithm. Algorithms 16 (8), 392.

[R104] Wallace L, Bi JZ, Ott WR, Sarnat J, Liu Y, 2021. Calibration of low-cost PurpleAir outdoor monitors using an improved method of calculating PM2.5. Atmos. Environ. 256, 118432.

[R105] Wallace LA, Zhao TK, Klepeis NE, 2022. Indoor contribution to PM2.5 exposure using all PurpleAir sites in Washington, Oregon, and California. Indoor Air 32 (9), 13105. 10.1111/ina.13105 36168225

[R106] Wang YW, Du YJ, Fang JL, Dong XY, Wang Q, Ban J, Sun QH, Ma RM, Zhang WJ, He MZ, Liu C, Niu Y, Chen RJ, Kan HD, Li TT, 2022. A random forest model for daily PM2.5 personal exposure assessment for a Chinese cohort. Environ. Sci. Technol. Lett. 9 (5), 466–472.

[R107] Wangchuk T, Mazaheri M, Clifford S, Dudzinska MR, He CR, Buonanno G, Morawska L, 2015. Children’s personal exposure to air pollution in rural villages in Bhutan. Environ. Res. 140, 691–698. 26087435 10.1016/j.envres.2015.06.006

[R108] Washington State Geospatial Portal Washington, 2010. State land use. https://geo.wa.gov/datasets/wa-geoservices::washington-state-land-use-2010/about. (Accessed 12 August 2024).

[R109] Wickham H, 2007. Reshaping data with the reshape package. J. Stat. Software 21 (12), 1–20.

[R110] Wyatt LH, Weaver AM, Moyer J, Schwartz JD, Di Q, Diaz-Sanchez D, Cascio WE, Ward-Caviness CK, 2022. Short-term PM2.5 exposure and early-readmission risk: a retrospective cohort study in North Carolina heart failure patients. Am. Heart J. 248, 130–138. 35263652 10.1016/j.ahj.2022.02.015 PMC9064928

[R111] Xiang JB, Austin E, Gould T, Larson T, Yost M, Shirai J, Liu YS, Yun S, Seto E, 2020. Using vehicles’ rendezvous for in situ calibration of instruments in fleet vehicle-based air pollution Mobile monitoring. Environ. Sci. Technol. 54 (7), 4286–4294. 32150678 10.1021/acs.est.0c00612 PMC13187943

[R112] Xiang JB, Hu LM, Hao JY, Wu SQ, Cao JP, Seto E, 2023. Characterization of time- and size-dependent particle emissions and decay from cooking oil fumes in residence: impacts of various intervention measures. Build. Simulat. 16 (7), 1149–1158.

[R113] Xu Y, O’Sharkey K, Cabison J, Rosales M, Chavez T, Johnson M, Yang T, Cho SH, Chartier R, Grubbs B, Lurvey N, Lerner D, Lurmann F, Farzan S, Bastain TM, Breton C, Wilson JP, Habre R, 2024. Sources of personal PM2.5 exposure during pregnancy in the MADRES cohort. J. Expo. Sci. Environ. Epidemiol. 34, 868–877. 38326532 10.1038/s41370-024-00648-z PMC11446843

[R114] Yang KQ, Liu NR, Weschler CJ, Weschler LB, Mo JH, Xu Y, Wei JY, Wang YM, Zhao ZH, Kan HD, Zhang YP, 2024. Maximizing the net economic benefits of regulating indoor air quality in China. Sustain. Cities Soc. 117, 105938.

[R115] Yi L, Xu Y, Eckel SP, O’Connor S, Cabison J, Rosales M, Chu D, Chavez TA, Johnson M, Mason TB, Bastain TM, Breton CV, Dunton GF, Wilson JP, Habre R, 2022. Time-activity and daily mobility patterns during pregnancy and early postpartum - evidence from the MADRES cohort. Spat Spatio-Temp. Epidemiol. 41, 100502. 10.1016/j.sste.2022.100502 PMC9198358 35691658

[R116] Zamora ML, Pulczinski JC, Johnson N, Garcia-Hernandez R, Rule A, Carrillo G, Zietsman J, Sandragorsian B, Vallamsundar S, Askariyeh MH, Koehler K, 2018. Maternal exposure to PM2.5 in south Texas, a pilot study. Sci. Total Environ. 628–629, 1497–1507. 10.1016/j.scitotenv.2018.02.138 PMC13041583 30045568

[R117] Zamora ML, Xiong FLZ, Gentner D, Kerkez B, Kohrman-Glaser J, Koehler K, 2019. Field and laboratory evaluations of the low-cost plantower particulate matter sensor. Environ. Sci. Technol. 53 (2), 838–849. 30563344 10.1021/acs.est.8b05174

[R118] Zeger SL, Thomas D, Dominici F, Samet JM, Schwartz J, Dockery D, Cohen A, 2000. Exposure measurement error in time-series studies of air pollution: concepts and consequences. Environ. Health Perspect. 108 (5), 419–426. 10811568 10.1289/ehp.00108419 PMC1638034

[R119] Zhai SR, Albritton D, 2020. Airborne particles from cooking oils: emission test and analysis on chemical and health implications. Sustain. Cities Soc. 52, 101845.

[R120] Zhang Y, Lin YP, 2019. An interactive method for identifying the stay points of the trajectory of moving objects. J. Vis. Commun. Image Represent. 59, 387–392.

[R121] Zhang L, Guo C, Jia X, Xu H, Pan M, Xu D, Shen X, Zhang J, Tan J, Qian H, Dong C, Shi Y, Zhou X, Wu C, 2018. Personal exposure measurements of school-children to fine particulate matter (PM2.5) in winter of 2013, Shanghai, China. PLoS One 13 (4), e0193586. 29608594 10.1371/journal.pone.0193586 PMC5880346

[R122] Zhang YQ, Yin ZX, Li SJ, Zhang JF, Sun HZ, Liu KY, Shirai K, Hu KJ, Qiu CX, Liu XY, Li YC, Zeng Y, Yao Y, 2023. Ambient PM2.5, ozone and mortality in Chinese older adults: a nationwide cohort analysis (2005–2018). J. Hazard Mater. 454, 131539. 37149946 10.1016/j.jhazmat.2023.131539 PMC11758737

[R123] Zusman M, Schumacher CS, Gassett AJ, Spalt EW, Austin E, Larson TV, Carvlin G, Seto E, Kaufman JD, Sheppard L, 2020. Calibration of low-cost particulate matter sensors: model development for a multi-city epidemiological study. Environ. Int. 134, 105329. 31783241 10.1016/j.envint.2019.105329 PMC7363217

